# Salicylic acid-primed defence response in octoploid strawberry ‘Benihoppe’ leaves induces resistance against *Podosphaera aphanis* through enhanced accumulation of proanthocyanidins and upregulation of pathogenesis-related genes

**DOI:** 10.1186/s12870-020-02353-z

**Published:** 2020-04-08

**Authors:** Jun Feng, Min Zhang, Kang-Ning Yang, Cai-Xia Zheng

**Affiliations:** College of Biological Sciences and Technology, Beijing Forestry University, Beijing, 100083 China

**Keywords:** Salicylic acid, Octoploid strawberry, *Podosphaera aphanis*, Proanthocyanidins, *PR* genes

## Abstract

**Background:**

*Podosphaera aphanis*, a predominately biotrophic fungal pathogen, causes significant yield losses of strawberry. China is the largest strawberry producer in the world, and selecting for powdery mildew-resistant cultivars is desirable. However, the resistance mechanism against *P. aphanis* in the octoploid strawberry remains unclear.

**Results:**

To understand possible mechanisms of disease resistance, we inoculated strawberry leaves with *P. aphanis*, and examined the expression profiles of candidate genes and the biochemical phenotypes in strawberry leaves of two groups. The unigenes obtained from ddH_2_O- and SA-pretreated leaves resulted in a total of 48,020 and 45,896 genes, respectively. KEGG enrichment showed that phenylpropanoid biosynthesis and plant hormone signal transduction pathways were enriched to a noticeable extent. DEG analysis showed that key TFs genes associated with the SA signaling pathway could play important role in the strawberry–*P. aphanis* interaction. In particular, *FaWRKY70*, *FaJAZ1* and *FaMYC2-like*, involved in regulating the antagonistic effect of SA and JA signaling pathway, leading to increased expression of SA-responsive genes (in particular *PR1*, *PR2*, *PR3*, and *PR5*) compared to a decline in expression of JA-responsive genes (*FaJAR1*, *FaAOS*, and *FaLOX2*). Furthermore, SA pretreatment induced accumulation of PAs by activating the MBW complex and inhibit powdery mildew growth.

**Conclusions:**

This study describes the role of the proanthocyanidins (PAs), pathogenesis-related (PR) genes, SA, and transcription factors in regulatory model against *P. aphanis*, which coincided with an early activation of defense, leading to the accumulation of PAs and the PR proteins.

## Background

Plants have evolved immune systems to defend against various pathogenic microorganisms, which rely on the recognition of potential effectors by both pathogen-associated molecular pattern-triggered and effector-triggered immunity [1]: the defence mechanisms include oxidative burst, reinforcement of the cell wall, production of pathogenesis-related (PR) proteins, and a rapid hypersensitive response (HR) at the penetration site [2]. Powdery mildew (PM) is a widespread fungal disease of most plants, caused by Ascomycetes [3], which are obligate biotrophic fungi that form haustoria for the uptake of nutrients. Over 400 PM species colonise nearly 10,000 angiosperm species [4], including monocotyledonous and dicotyledonous plants [5]. Previous plant PM research has focused on major resistance genes involved in signalling pathways and secondary metabolites, especially in economically important crops, such as tomato, barley, apple, and wheat, as well as in the reference species *Arabidopsis* [6]. Therefore, the study of molecular resistance mechanisms is vital to improve the quality and yield of economically important crops.

Emerging evidence has shown that phytohormones, especially salicylic acid (SA) and jasmonic acid (JA), and ethylene (ET) signalling pathways regulate plant defence against various pathogens [7]. Upon biotrophic pathogen attack, the accumulation of endogenous SA induces expression of several PR genes to enhance resistance. The SA- and JA/ET-mediated defence pathways are often mutually antagonistic [8]. When SA levels increase, NPR1 oligomers dissociate into monomers, which then enter the nucleus and interact with TGA transcription factors (TFs) [9] and TGA-interacting GLUTAREDOXIN 480 (GRX480), which regulates SA/JA antagonism [10]. WRKY70 is also required for expression of pathogenesis-related protein (PR1) and is a key regulator of SA/JA antagonism [11]. In *Arabidopsis*, SA-mediated defence plays a vital role against biotrophs, while JA/ET defends against necrotrophs [12]. Orthologues of genes involved in SA–JA crosstalk include NPR1, WRKY70, GRX480, MYC2, and JAZs [13]. SA/JA pathway antagonism interacts with other phytophormones (such as ET and gibberellic acid), regulating trade-offs between biotrophs and necrotrophs.

The octoploid strawberry (*Fragaria × ananassa*) is a perennial plant belonging to *Rosaceae* [14]. Strawberry is widely cultivated in China and is reproduced asexually. *Podosphaera aphanis* is a biotrophic fungal disease of strawberry [15] that results in considerable losses in production and is deemed to be one of the most destructive diseases. Due to the large-scale promotion of Japanese varieties (especially new varieties derived from Benihoppe) and suitable environmental conditions in winter greenhouses, powdery mildew has become serious disease in China [16]. Despite the availability of total genome sequence information for octoploid strawberry [17], its mechanisms of defence against *P. aphanis* at the molecular level remain to be clarified. Transcriptome analysis has shown differentially expressed genes (DEGs) related to secondary metabolism, signal transduction, and disease resistance were upregulated and played crucial roles in the early defence against *P. aphanis* [18]. Furthermore, functional identification of candidate genes from the diploid strawberry has enabled the investigation of resistance to *P. aphanis*, including *FvHsfB1a* [19], *FvMLO* [20], and *FvWRKY42* [21]. Antisense expression of PpMlo1-conferred resistance in the octoploid strawberry to *P. aphanis*, indicating that the Mlo-based resistance mechanism is functional in strawberry [22]. Moreover, ectopic expression of *AtNPR1* in diploid strawberry showed enhanced resistance to *P. aphanis*, suggesting that NPR1 confers broad-spectrum disease resistance [23]. Overexpression of *AtELP3* and *AtELP4* in diploid strawberry conferred enhanced resistance to *P. aphanis*, suggesting that *ELP* genes may confer resistance against PM [24]. Recent advances in our understanding of resistance against *P. aphanis* have revealed that it is polygenic and quantitatively inherited [25]. Despite these efforts, little research has focused on the molecular resistance mechanisms of the octoploid strawberry. Most studies have focused on applied research, and particularly on pesticides used in practice. The intensive use of fungicides for disease control can be hazardous to the environment and human health [26]. Therefore, recent attention has focused on gaining a better understanding of the resistance mechanisms of the octoploid strawberry against *P. aphanis*.

In this study, we first investigated the fluorescence parameters and the germination percentage of conidia to determine infection time. Next, we analysed the transcriptome of leaves during different infection stages in two groups (ddH_2_O-treated and SA-treated). KEGG showed that DEGs were mainly involved in phenylpropanoid and flavonoid biosynthesis, and hormone signalling transduction. Moreover, we also detected dynamic patterns of total flavonoid content (TFC), as well as PAs and SA content. Furthermore, we analyzed the phylogenetic tree and conserved sequence to identify highly homologous proteins, which were used to analyse the correlation between these key genes through transcript level comparison. Our results provide insight into the molecular level underlying the defence mechanism of the octopliod strawberry response to *P. aphanis*. SA, PAs, TFC, and signalling molecules are also potential regulatory compounds involved in SA-induced resistance to *P. aphanis*. This study is the first to characterise the resistance mechanisms against *P. aphanis* in the octoploid strawberry.

## Results

### Leaf dynamics in response to *P. aphanis*

Changes in fluorescence parameters and morphology were determined in a greenhouse environment following inoculation with *P. aphanis*. In October, natural day light was only 8–9 h/day. Temperatures were approximately 18–28 °C during the day and 7–14 °C at night, while the relative humidity were approximately 39–58 °C during the day and 91–95 °C at night throughout the experiment (Figure S1). According to Dodgson et al., (2007) 97–100% relative humidity is optimum for germination of condia, while optimum temperature for germination is 15–25 °C and optimum temperature for sporulation is 20 °C [27]. Therefore, the temperature and humidity in Greenhouse are suitable for *P. aphianis* germination.

**Fig. 1:**
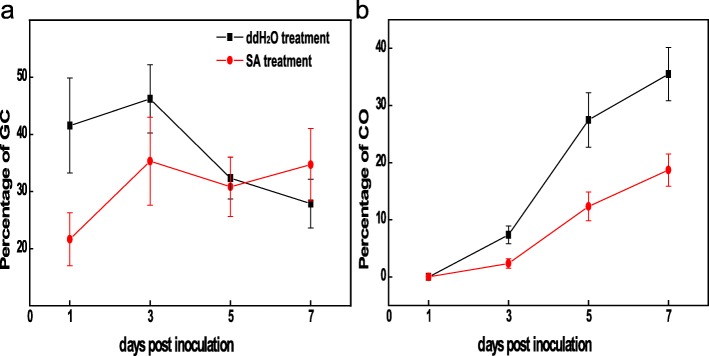
Percentage of *P. aphanis* conidial germination on ddH_2_O-treated and SA-treated strawberry leaves. GC, germinated conidia without forming appresoria; CO, germinated conidia with conidiophore. Data are means of five replicates ± standard deviation. Different letters at each time point represent significant differences among treatments at *P* < 0.05. dpi: days post inoculation

**Fig. 2:**
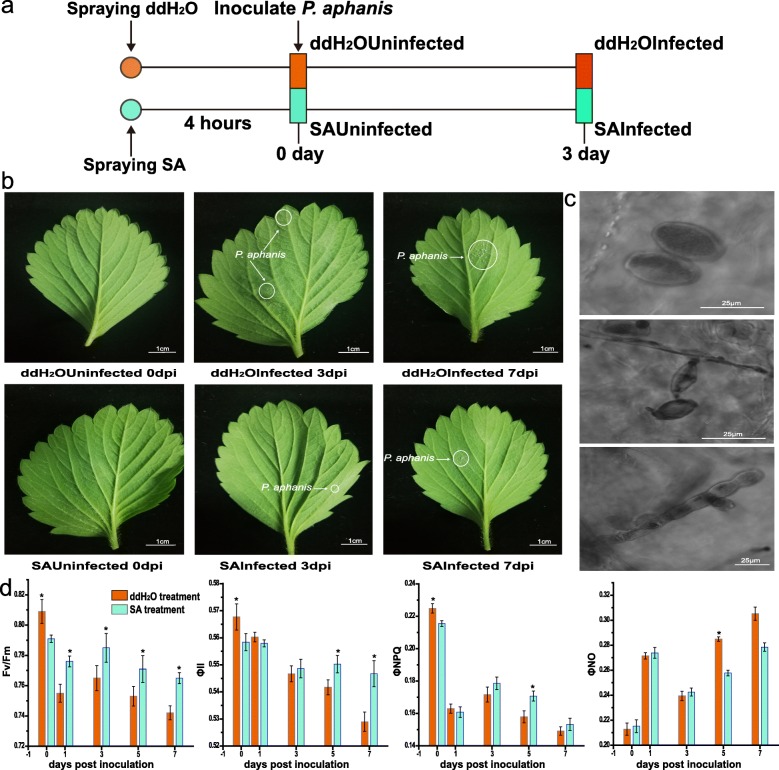
Pretreatment with salicylic acid (SA) reduces the colonisation area of *P. aphains* in the leaf of strawberry. **a** Experimental outline. Young strawberry leaves were sprayed with distilled water and SA. After 4 h, a subset of plants from each group were inoculated with *P. aphains*. Leaves were collected from 10 plants at 0 dpi and 3 dpi after *P. aphanis* inoculation. **b** Photographic documentation of the progression of *P. aphanis* infection in strawberry leaves treated with ddH_2_O or SA at different time points after inoculation (0, 3, and 7 dpi). Bar = 1 cm. **c***P. aphanis* conidial germination on strawberry leaves at different time points. **d** Time course of strawberry leaf responses to infection with *P. aphanis*. Changes in the chlorophyll fluorescence parameters *F*_*v*_*/F*_*m*_, *Φ*_*II*_, *Φ*_*NPQ*_, and *Φ*_*NO*_ were measured in leaves inoculated with *P. aphanis*. Measurements were taken at 0, 1, 3, 5, and 7 days post-inoculation (dpi). *F*_*v*_*/F*_*m*_ was determined for 20 min dark-adapted leaves following initial exposure of plants to a saturating light pulse. All values are expressed as mean ± standard error; *N* = 3 for *F*_*v*_*/F*_*m*_, *Φ*_*II*_, *Φ*_*NPQ*_, and *Φ*_*NO*_ measurements

To investigate leaf resistance in response to *P. aphanis*, the conidial germination percentage of *P. aphanis* was recorded over the entire experimental period (Fig. 1). Visual symptoms (white mycelium) began appearing on leaves at 3 dpi in both groups, and obvious differences were observed between the two groups (there was a larger disease area in ddH_2_O treatment than SA treatment). Whereas extensive colonisation occurred along the leaf in the ddH_2_O group at 7 dpi, only small, restricted colonisation was observed in the SA group (Fig. 2b). The developmental stages of *P. aphanis* were also observed using a microscope (Fig. 2c).

To further ascertain the optimum sampling time, chlorophyll fluorescence parameters in both groups infected with *P. aphanis* were detected (Fig. 2d). *F*_*v*_*/F*_*m*_ is the state of the initial part of photosynthesis and reflects the degree of plants under stress. *Φ*_*PSII*_ is the actual PSII efficiency and reflects the current actual light energy conversion efficiency of the photosynthetic mechanism. *Φ*_*NPQ*_ is the energy dissipated as heat through a regulated photoprotection mechanism, if the value is higher, indicating plant has a higher photoprotection ability. *Φ*_*NO*_ is the passive dissipation of heat and fluorescent energy, suggesting the self-protection ability under excessive light is lost. Both groups showed decrease in the *F*_*v*_*/F*_*m*_ ratio at 1 dpi and an increase at 3 dpi, followed by a continuous decline in *F*_*v*_*/F*_*m*_ values from 3 to 7 dpi. The *F*_*v*_*/F*_*m*_ ratios in the SA group were higher than those in the ddH_2_O group from 1 to 7 dpi, and coincided with visual symptoms at the corresponding time points (Fig. 2d). *Φ*_*PSII*_ showed a continuous decline over the course of the experiment; *Φ*_*PSII*_ and *Φ*_*NPQ*_ were higher in the SA group than ddH_2_O group from 3 to 7 dpi. In contrast to *F*_*v*_*/F*_*m*_, *Φ*_*PSII*_, and *Φ*_*NPQ*_, *Φ*_*NO*_ in the ddH_2_O group was higher than that in the SA group from 5 to 7 dpi. Lower *F*_*v*_*/F*_*m*_ values usually corresponded to higher *Φ*_*NPQ*_ values, indicating dissipative processes that lowered the PS photochemical efficiency. *F*_*v*_*/F*_*m*_ values was lower in ddH_2_OInfected than in SAInfected, suggesting that there was damage to photosystem II caused by *P. aphains* at 3 dpi. However, there was no significant difference in the value of *Φ*_*NO*_ between the two treatments expect 5 dpi (Fig. 2d). Infected with *P. aphanis*, reductions in *F*_*v*_*/F*_*m*_, *Φ*_*PSII*_, and *Φ*_*NPQ*_ were detected at 3 days, which was consistent with the appearance of visible symptoms. Therefore, these divergent phenotypes (3 dpi) were used in the transcriptome analyses.

### Changes in the transcriptome in strawberry leaves infected with *P. aphanis*

To determine the transcriptome profile of strawberry in response to *P. aphanis*, RNA sequencing (RNA-Seq) analyses were performed on 12 samples (ddH2OInfected, ddH2OUnifected, SAInfected, and SAUnifected, three replicates per treatment) (Fig. 2a). Approximately 600 million raw reads were obtained in total, the clean reads were mapped to the *F. ananassa_Camarosa* genome (Table S1). FPKM values of DEGs were used to calculate the fold changes of ddH_2_OInfected/ddH_2_OUnifected and SAInfected/SAUnifected. Principle component analysis (PCA) showed that PC1 and PC2 could explain 64.40% of the total transcript expression level variance, in which PC1 explained 50.96% of the total detected variation according to genotype, while the PC2 separated samples according to treatment and explained 12% of the variance (Figure S2). There was greater separation on PC2 for samples from the SAInfected and SAUninfected genotype, indicating a stronger transcriptional differentiation during SA-pretreatment.

Among the two groups, 48,020 transcripts and 45,896 transcript genes were expressed in the strawberry leaves from ddH_2_OInfected/ddH_2_OUnifected and SAInfected/SAUnifected leaves, 43,103 genes were commonly expressed in ddH_2_OInfected/ddH_2_OUnifected leaves, 2770 genes were expressed only in ddH_2_OUnifected leaves, and 2147 genes were expressed only in ddH_2_OInfected leaves. While SAInfected/SAUnifected had 41,939 commonly expressed genes, 1755 genes were expressed only in SAUnifected and 2202 in SAInfected leaves (Figure S3a). Based on p-adjust < 0.05 and |log2FC| ≥ 1, a total of 4417 and 3754 DEGs were detected in ddH_2_OInfected/ddH_2_OUnifected and SAInfected/SAUnifected leaves, respectively. As indicated in Figure S2ab, 2224 genes were upregulated in SAInfected/SAUnifected leaves compared to 2110 in ddH_2_OInfected/ddH_2_OUnifected (log2FC ≥ 1 for upregulated). By contrast, a greater number of genes were downregulated genes in ddH_2_OInfected/ddH_2_OUnifected (2088) compared to SAInfected/SAUnifected leaves (1530) (log2FC ≤ − 1 for downregulated). However, there were commonly 921 genes expressed at two time-points. A great number of DEGs (upregulated of 1476 genes in ddH_2_O treatment, upregulated of 1565 genes in SA treatment, downregulated of 1801 genes in ddH_2_O treatment, downregulated of 1268 genes in SA treatment) were noticeable in both groups, indicating that DEGs in response to *P. aphanis* varied greatly (Fig. S3d). Based on BIRCH clustering, a total of 20 clusters of expression profiles were identified with distinguishable expression patterns during strawberry-*P. aphanis* interaction in ddH_2_O and SA treatment (Figure S3e). The powdery mildew infection lead to the rapid up- or down-regualtion of transcripts in both ddH_2_O treatement (cluster 1, 4, 6, 3, and 5) and SA treatment (cluster1, 4, 6, 2, 3, and 5), indicating transcripts involved in fungus responses. Cluster7, cluster8, cluster9, and cluster10 showed irregular changes in both treatments. In general, the results demonstrated that DEGs in the ddH_2_O treatemnt were delayed compared with the SA treatment.

Twenty enriched GO terms were mainly categorised as biological process and molecular function (Figure S4ab). In the ddH_2_O-treated group, most genes were involved in oxidation-reduction process, metal ion binding, cation binding, and oxidoreductase activity. By contrast, only single-organism metabolic process, oxidation-reduction process, and oxidoreductase activity were associated with the SA-treated group. KEGG enrichment analysis showed some of the same pathways, such as phenylpropanoid biosynthesis, phenylalanine metabolism, and flavonoid biosynthesis (Figure S4cd). Enrichment pathway under both groups was plant hormone signal transduction (Figure S5), indicating that SA and JA signalling pathway were mainly involved in the response to *P. aphanis*. We concluded that the genes for phenylpropanoid and flavonoid biosynthesis, and TFs involved in hormone signal transduction played an important role in strawberry defence aganist *P. aphanis*.

### The flavonoid biosynthesis pathway participates in resistance against *P. aphanis*

**Fig. 3:**
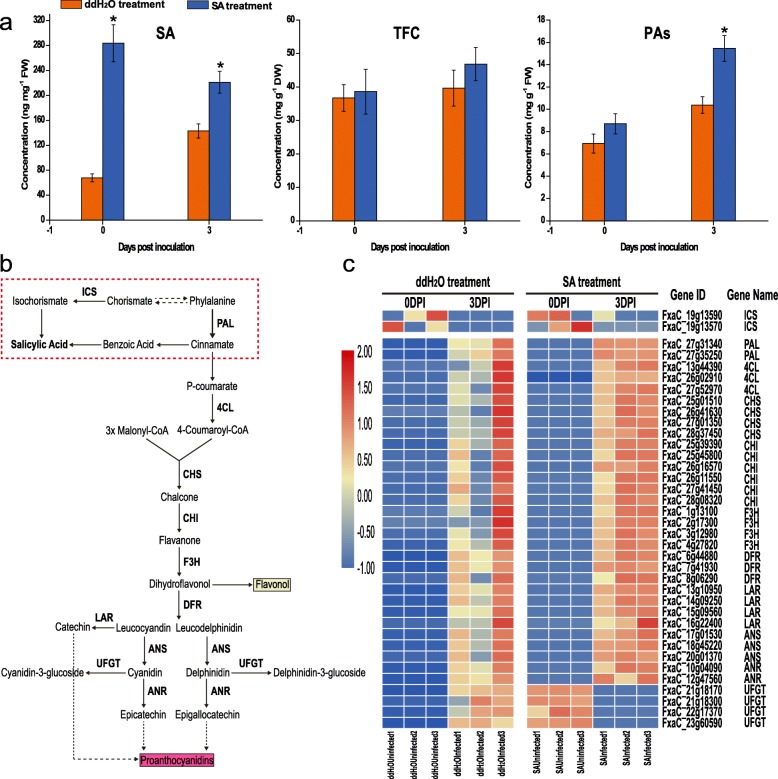
Salicylic acid (SA) content, total flavonoid content (TFC), and proanthocyanidins (PAs) in P. aphanis-infected strawberry leaves in the ddH_2_O-treated and SA-treated groups. **a** Concentration of SA, TFC, and PAs in strawberry leaves with or without SA treatment at two different infection time. Data are expressed as the mean ± standard deviation (SD) of three biological replicates. Different letters above the bar indicate statistically significant differences between groups at the same time point (P < 0.05). **b** Flavonoid pathway results in the formation of PAs. **c** Relative expression of PA biosynthesis genes. Heatmap was generated using the average values [log10 (FPKM+ 1)] of three biological samples per treatment

To determine whether exogenous SA could trigger PA accumulation upon *P. aphains* attack, TFC and PA metabolites were measured (Fig. 3a). The SA concentration in the SA-treated group was significant higher than that in the ddH_2_O-treated group at two infection points. Moreover, there is no significant difference in TFC content between two groups, although the value was higher in the SA-treated group than in the ddH_2_O-treated group. Additionally, PA levels were significant higher in the SAInfected than in the ddH_2_OInfected. To further clarify the regulatory mechanism of SA-triggered the accumulation of PAs, the regulation of DEGs associated with phenylpropanoid and flavonoid pathways was investigated (Fig. 3c). RNA-Seq showed the upregulated expression of key genes involved in the flavonoid pathway. Transcript levels of 4-coumarate-CoA ligase 2 (4CL) encoding genes, chalcone synthase (CHS) encoding genes, chalcone isomerase (CHI) encoding genes, flavanone 3-hydrolase (F3H) encoding genes, dihydroflavonol reductase (DFR) encoding genes, leucoanthocyanidin reductase (LAR) encoding genes, anthocyanidin synthase (ANS) encoding genes, and anthocyanidin reductase (ANR) encoding genes were increased at 3 dpi compared with 0 dpi in both groups. Moreover, the expression levels of these genes were also higher in the SA-treated group than that in the ddH_2_O-treated group (Table S4). Compared with increased expression of UDP-glucose: anthocyanidin: flavonoid glucosyltransferase (UFGT) encoding genes in the ddH_2_OInfected, the expression of UFGT in the SAInfected was markedly downregulated, indicating that SA could suppress UFGT to produce more PAs. Overall, we suggest a potential role of PAs in enhancing resistance against *P. aphanis*. Therefore, we propose that TFC and PAs are potentially important antifungal compounds in defence against *P. aphanis*.

### The MBW complexes involved in PA biosynthesis induced upon *P. aphains*

**Fig. 4:**
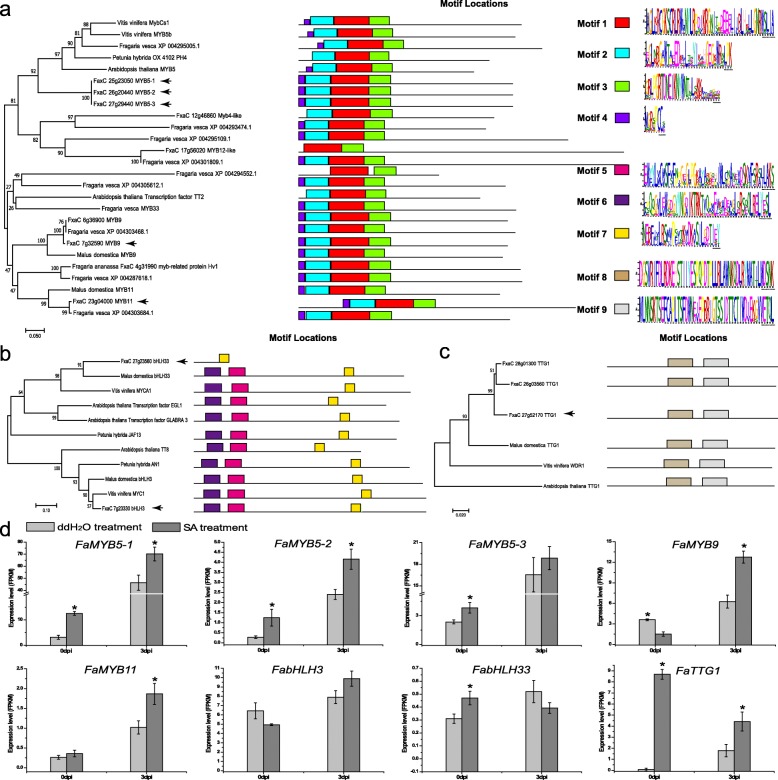
Identification of putative transcriptional regulator involved in proanthocyanidins (PAs) biosynthesis in strawberry leaves infected by *P. aphanis*. The phylogenetic relationships existing between the strawberry MYB (**a**), basic helix–loop–helix (bHLH) (**b**) and TRANSPARENT TESTA GLABRA1 (TTG1-like) (**c**) proteins used in this study (black arrow) with their closest homologs. **d** Changes in relative expression of FaMYB5, FaMYB9, FaMYB11, FabHLH33 and FaTTG1 genes at 0 dpi and 3 dpi under ddH_2_O-treatment and SA-treatment. The values were normalized against controls and correspond to the mean of three biological replicates ± S.E. Asterisks indicate significant differences (* *p* < 0.05)

Previous study showed that FaMYB9/FaMYB11, FabHLH3 and FaTTG1 functionally interact to regulate PA synthesis during strawberry fruit development [28]. Phylogenetic analysis showed that *FaMYB5–1*, *FaMYB5–2*, and *FaMYB5–3* are most similar to *AtMYB5*. R2R3-MYB motif analysis showed that motif 1 and motif 2 were R2 conserved domain, while motif 3 was R3 conserved domain (Fig. 4a). In addition, another two R2R3-MYB, *FaMYB9* and *FaMYB11* are homologues of *MdMYB9* and *MdMYB11*. Interestingly, phylogenetic analysis shows that the identified proteins were similar in length to their homologues, expect for *FabHLH33* (FxaC_27g23560). Motif analysis also shows that there is missing motif 5 (the bHLH domain) and motif 6 (the ACT-like dimerization domain) in FabHLH33, which is more closely related to MdbHLH33 and VvMYCA1 than to AtEGL1/AtEGL3 (Fig. 4b). Besides, FabHLH3 is similar to VvMYC1, MdbHLH, PhAN1 and AtTT8. FaTTG1 showed the high similarity to MdTTG1 and all identified proteins had two WD40 domains (motif 8 and motif 9) (Fig. 4c).

The expression pattern analysis of key genes (*FaMYB9*, *FaMYB11*, *FabHLH3*, *FabHLH33*, and *FaMYB5*) encoding the MBW complex were performed to understand the differences in TFC and PAs in two treatments (Fig. 4d). The expression pattern of the *FaMYB5*, *FaMYB9*, *FaMYB11*, *FabHLH3* and *FaTGG1* had same trend, with the higher expression level in SAInfected compared to ddH_2_OInfected (Fig. 4d), which may be related to PAs accumulation pattern in the same point (Fig. 2a). In turn, *FabHLH3* does not show significant expression level at two point under two groups, although SA treatment also increased its level at 3 dpi compared to the inhibition of its level at 0 dpi. The significant differences in *FabHLH33* between treatments were observed at 0 dpi, and then SA treatment further decreased its level at 3dpi. In the case of *FaTTG1*, a high expression was observed in SAUnifected.

### The SA biosynthesis and SA signalling pathway contributes to enhanced resistance

SA biosynthesis occurs in two distinct pathways; the phenylalanine pathway and the ICS1 pathway, from which nearly 95% of SA is produced [29]. The ISC1 pathway involves two steps: the conversion of chorismate to isochorismate, which is first catalyzed by ICS1, followed by the conversion of isochorismate to SA by an unknown enzyme [30]. For the phylogenetic analysis, highly homologous proteins of strawberry were identified (Figure S5a-e). In this study, gene encoding ICS1 were downregulated in both groups (Fig. 3d and Figure S6), while two PAL genes showed strong upregulation. Additionally, expression analysis showed *FaICS2* with very low expression but high expression in *FaPAL*. In general, these results suggested that the SA biosynthesis of strawberry might be mainly from PAL pathway. Moreover, EDS1 and PAD4 act upstream of SA accumulation at the infection site, while expression of the EDS1/PAD4 complex can be increased by exogenous SA [31]. Compared with 0 dpi, *FaPAD4* showed upregulation in both groups at 3 dpi (Figure S5e). No obvious change in *FaEDS1* was observed between ddH_2_OInfected and ddH_2_OUninfected, whereas SA induced strong expression of *FaEDS1* at two infection points in SA-treated group, which suggests *P. aphanis* might suppress EDS1 to inhibit SA biosynthesis. Interestingly, no significant difference in *FaPAD4* between two groups was observed, indicating that EDS1 might paly crucial role in mediating SA accumulation in strawberry.

**Fig. 5:**
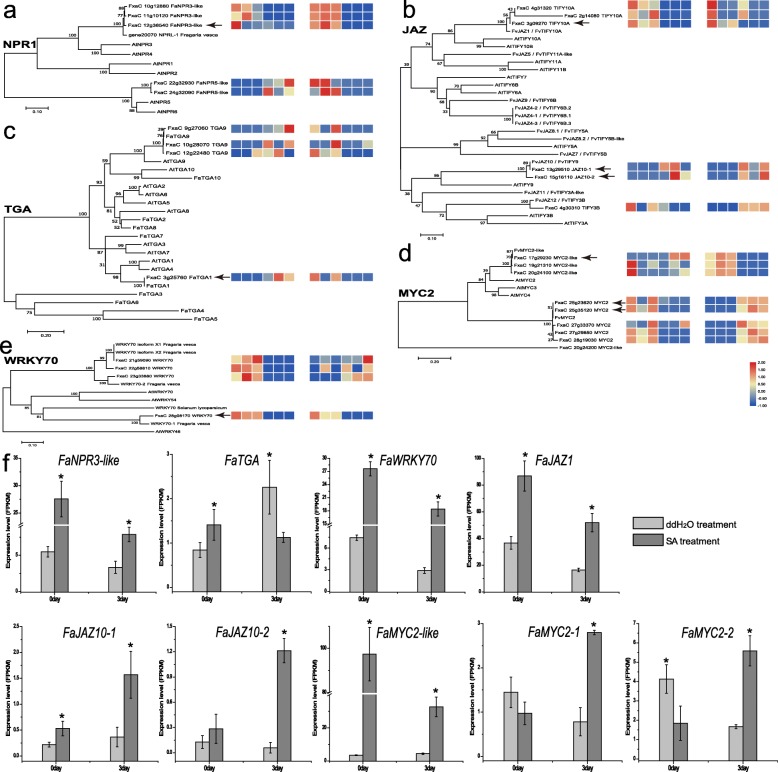
Identification of putative *DEGs* genes JA biosynthesis in strawberry leaves infected by *P. aphanis*. The phylogenetic relationships existing between the strawberry NPR (**a**), JAZ (**b**), TGA (**c**), MYC2 (**d**), and WRKY70 (**e**) proteins used in this study (black arrow) with their closest homologues. Numbers at branches indicate posterior probabilities and bootstrap percentages based on 1000 replicates. Heatmap was generated using the average values [log10 (FPKM+ 1)] of three biological samples per treatment, representing the relative transcript expression levels of DEGs in leaves. The colour bar indicates the expression values as an increasing intensity gradient (blue, low expression; red, high expression). **f** Changes in relative expression of *FaNPR3-like*, *FaTGA*, *FaJAZ1*, *FaJAZ10*, *FaMYC-like* and *FaMYC2* genes at 0 dpi and 3 dpi in both groups. The values were normalized against controls and correspond to the mean of three biological replicates ± S.E. Asterisks indicate significant differences (* *p* < 0.05). At, *Arabidopsis thaliana*, all protein information are available at Uniprot database (https://www.uniprot.org/). NPR, Non-expressor of pathogenesis-related gene; JAZ, JASMONATE ZIM-Domain; TGA, TGACG motif-binding factor; WRKY, W box (5′-(T) TGAC [CT]-3′) motif-binding factor; MYC2, nuclear-localized basic helix-loop-helix–type transcription factor

In the transcriptional reprogramming outline of transcripts associated to strawberry resistance to *P. aphains* and the accumulation of SA content indicates that genes for SA biosynthesis and TFs involved in SA signaling, play a pivotal importance. In this study, they were further characterised in terms of phylogeny analysis and expression pattern aiming to dissect their functional roles in the *P. aphains* response in strawberry. There are DEGs across the two treatments, such as NPR, TGA, WRKY, MYC2 and JAZ, which are involved in signal transduction (Figure S5). As expected, as SA and JA pathways are activated in both groups, require large scale transcriptional reprogramming, especially transcription factors. To further identify a possible role for these DEGs in the regulation of defence responses, using protein sequence of these DEGs, unrooted phylogenetic trees were constracted to show evolutionary relationships between F. ananssna and *A. thaliana* (Fig. 5).

Non-expressor of pathogenesis-related gene 1 (NPR1) is positive regulator of the SA-dependent signaling pathway, and then mediates the binding of TGA factors to the as-1 motif found in the pathogenesis-related PR-1 gene. *FaNRP3-like* was phylogenetically closer to *FvNPRL-1*, which was more similar to *AtNPR3/AtNPR4* than *AtNPR1*. *FvNPRL-1* likely functions similar to Arabidopsis NPR3/NPR4 as a negative regulator of the SA-mediated defense [32]. As shown in Fig. 5, the strong induction of *FaNPR3-like* was detected as early as 4 h after SA treatment, then decreased rapidly in both groups, suggesting that *FaNPR3-like* (similar to *FvNPRL-1*) was a repressor to quickly balance the effect caused by excessive SA. Therefore, *FaNPR3-like* likely acts as negative regulators of SA-mediated defence pathway. As an important co-activator of NPR1, *FaTGA* showed a contrasting expression profile between ddH_2_OInfected/ddH_2_OUninfected and SAInfected/SAUninfected in this study (Fig. 5). FaTGA shared high similarity to FaTGA1, which might interact with FaNPR1 and play a crucial role in the response to powdery mildew disease [33]. Furthermore, FaTGA also showed closly evolutionary relationships with AtTGA, which was shown to regulated SA biosynthesis by modulating *SARD1* [29]. Thus, FaTGA1 likely function as positive regualtors of SA biosynthesis and SA-mediated defence. It is clear that WRKY70 is a downstream regulator of NPR1 and COI1 in positively mediating SA-dependent genes, compared to negatively regulate JA-responsive genes [30]. Although upregulation trend of *FaWRKY70* (similar to *SlWRKY70* and *AtWRKY70*) expression was observed in both groups, the transcript level of *FaWRKY70* was significantly higher in SA-treated group than in ddH_2_O-treated (Fig. 5), indicating that SA mediates *FaWRKY70* accumulation, which is consistent with previous studies [31, 34]. Because AtWRKY70 is a negative regulator of JA-responsive genes, we hypothesized that FaWRKY70 orthologs maybe a positive regulator of SA-responsive genes.

### DEGs involved in JA-dependent defence pathways

It is well known that the JA-SA crosstalk results in fine-tune plant’s defence against different pathogens [35]. The JAZ (JASMONATE-ZIM DOMAIN) proteins are key transcriptional repressors regulating various biological processes. As key repressors in JA responses, when JA-Ile levels are low, JAZ binds to MYC2, leading to repression of JA-responsive genes [36]. The JA signalling pathway could trigger PAs biosynthesis at early stages of strawberry fruit development, especially *FaMYC2*, *FaJAZ1* and *FaJAZ8.1*, which are JA-responsive genes and correlated with the activation of JA-Ile biosynthesis [37]. Although *FaJAZ1* (similar to *FvJAZ1*) displayed a reduction pattern during infection in both groups, a pronounced expression of *FaJAZ1* induced by SA at two points was observed (Fig. 5), according to PAs accumulation during SA-treated leaves response to *P. aphains*, indicating that exogenous SA could induce expression of JA biosynthesis genes at the early stage of infection, leading to accumulation of PAs to defence against fungus. In contrast, the expression of *FaJAZ10–1* and *FaJAZ10–2* showed a similar increment pattern from 0 to 3 dpi in both groups. The evolutionary relationship showed that *FaMYC2-like* had the highest identify with *FvMYC2-like*, *FaMYC2–1* and *FaMYC2–2* were clustered closely to *FvMYC2* [37]. *FaMYC2–1* and *FaMYC2–2* showed an expression increment in SA-treatment during infection in a similar way to that observed for *FaJAZ10–1* and *FaJAZ10–2* (Fig. 5). Collectively, a significant greater expression level was observed for *FaJAZ1* and *FaMYC2-like* in SA-treatment than that in ddH_2_O-treatment. Moreover, higher relative expression levels were observed for *FaMYC2-like* and *FaJAZ1* in SAUnifected. The expression pattern of genes encoding for key enzymes involved in JA biosynthesis such as *FaJAR*, *FaAOS*, and *FaLOX2* were also analyzed (Figure S6). At 0 dpi, SA-treated leaves exhibited higher transcript levels of these genes, which decreased from 1 to 3 dpi, expect *FaLOX2* (Figure S6). Interestingly, expression of *FaLOX2* in SAUninfected was higher than in ddH_2_OUninfected; however, at 3 dpi, no significant difference for *FaLOX2* between two groups was observed. Expression of *FaAOS* and *FaJAR1* showed similar patterns across all time points. Furthermore, these genes were highly homologous to those in previously studied [38, 39]. Taken together, key genes involved in JA biosynthesis followed the same downregulation pattern, which is in agreement with an antagonistic relationship between SA and JA pathway. Overall, *JAZ1*, *JAZ10–2*, *MYC2-like*, *MYC2–1*, and *MYC2–2* and other JA-signalling related genes (*FaLOX2*, *FaAOS*, and *FaJAR*) are downregulated during *P. aphanis* infection, compared to SA induced higher expression.

### The expression pattern of pathogen-related resistance genes

**Fig. 6:**
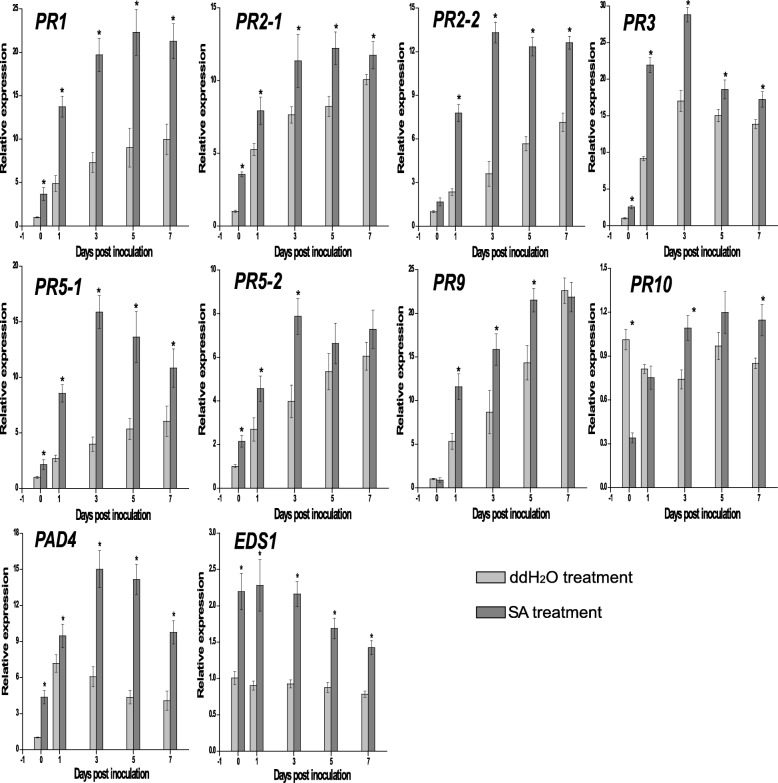
Changes in relative expression levels of pathogenesis-related (PR) genes, FaPAD4, and FaEDS1 at different time points under ddH_2_O treatment and SA treatment. The expression data were determined by RT-qPCR. The expression of two actin genes were used as reporter genes, and the values were normalised to the non-inoculated untreated control. Data are expressed as the mean ± SD of three biological replicates. An * above the bar indicates statistically significant differences between groups at the same time point

To investigate the defence response involved in SA-induced resistance, the major transcriptional changes (log2 fold change > 3) in response to *P. aphanis* infection between the SAInfected and SAUninfected were monitored (Table S5), including expression of *PR1*, *PR2* (endo-1,3-glucanase), *PR3* (chitinase), *PR5* (thaumatin-like protein), *PR9* (plant peroxidase), and *PR10*. Expression patterns of the *PR* genes in both groups were further examined by RT-qPCR (Fig. 6). The expression of these nine PR genes in strawberry leaves in SA treatment showed a similar trend as that observed with RNA-Seq. SA had a direct influence on *FaPR1*, *FaPR2*, *FaPR3*, *FaPR5*, and *FaPR10*, as evidenced by the significant difference in expression in the SA-treated group compared with the ddH_2_O-treated group. Moreover, during the experimental period, the expression of all *PR* genes continued for 5 days. In response to *P. aphanis*, the SA treatment exhibited significant higher expression of *FaPR1*, *FaPR2–1*, *FaPR2–2*, *FaPR3*, *FaPR5–1*, and *PR5–2* expression than that in the ddH_2_O treatment. These genes were also significant upregultated (1 dpi) prior to *P. aphanis* infection (white mycelium visible to the naked eye, 3dpi). These results suggested that *FaPR1*, *FaPR2–1*, *FaPR2–2*, *FaPR3*, *FaPR5–1*, and *PR5–2* might play direct defence against *P. aphanis*.

## Discussion

SA is called o-hydroxybenzoic acid and is a simple phenolic compound, which has been shown to induce resistance via the activation of plant defence response. It is also well known exogenous SA protects wheat against powdery mildew. Concerning the expensive cost of BTH, SA induces strawberry defence responses against *P. aphains* is an alternative in this study. Although comparative transcriptome data has provided insights into the diploid strawberry defence against *P. aphanis*, the molecular mechanisms in octoploid strawberry are still not well understood. Here, we have utilized the ddH_2_0-treated and SA-treated plants in response to *P. aphains* to identify potential key components involved in defence using a comparative transcriptome approach.

### Distinctive phenotypes of the two strawberry groups

In the present study, the *F*_*v*_*/F*_*m*_ decrease in ddH_2_OInfected was more significant than that of SAInfected, which clearly reflected that inhibition of PSII function was more serious infected by pathogen without SA-pretreatment, indicating that SA could reduce the damage of *P. aphains* and improve the original light energy conversion efficiency of PSII. Moreover, no significant change in *Φ*_*II*_ of leaves between ddH_2_OInfected and SAInfected was obersved, whereas SA treatment showed higher value compared with that in ddH_2_O treatment from 5 to 7 dpi, SA could alleviate the lea*ves.* Furthermore, *Φ*_*NPQ*_ of SAInfected was higher than that of ddH2OInfected, which suggested that SA could improve the non-radiative dissipation ability of strawberry leaves. Thus, *F*_*v*_*/F*_*m*_ and *Φ*_*NPQ*_ could be used as biomarker. Besides, SAInfected exhibited smaller area of infection that ddH_2_OInfected, suggesting that exogenous SA could enhance resistance to *P. aphains* and alleviate the disease process, without inhibiting the spread of powdery mildew. Of the KEGG pathways identified in two groups, the flavanol biosynthesis pathway was the most enriched, indicating the importance of gene regulation of the flavanol metabolite in octoploid strawberry in response to *P. aphanis*. Previous work has shown that genes related to secondary metabolism, signal transduction, transcription factors and disease resistance play important role in defense against *P. aphanis* [18].

### SA biosynthesis genes may contribute to resistance against *P. aphanis*

In *Arabidopsis*, the primary pathway for SA biosynthesis is the ICS1 pathway [34], which also relies on genes, such as EDS1 and PAD4. This pathway triggers early plant defence and the HR independently of PAD4, after which it recruits PAD4 to potentiate plant defence through the accumulation of SA. In ddH_2_O treatment *FaICS1* was down-regualtaed, while no change of this genes in the SA treatment was detected (Fig. 3c). Furthermore, the significant upregulation of *FaPAL* in both groups was observed (Fig. 3c), suggesting that strawberry might synthesize SA mainly through the PAL pathway. In strawberry, FaPAD4 and EDS1 may form a complex to regulate the defense system. No different of *FaPAD4* in two groups was observed (Figure S6f), we conclude that FaEDS1 is probably the important mediator in strawberry innate immunity. Higher resistance to *P. aphanis* SAInfected was also observed (Figs. 1ab and 2b). SA levels remained stable in ddH_2_O treatment in accordance with no slight changes in transcript accumulation of *FaPAL*, *FaEDS1*, and *FaPAD4*, indicating that *P. aphanis* could suppress these key genes to infect successful. During whole experiment, for these three genes, a steady decrease in transcript levels was observed in both treatment compared with the SA-biosynthesis genes, suggesting that SA pathway may play a crucial role in defense against *P. aphains*. Collectively, these results indicate that strawberry activates the defence system against *P. aphains* through SA biosynthesis.

### Increased PAs in the SA-primed leaves may restrict *P. aphanis*

It was recently reported that flavan was induced in the chemical defence against fungal [35, 36]. In this study, we showed that PA level and TFC increased in the SA treatment (Fig. 3a). SA increased PA accumulation in *Cistus heterophyllus* and in grapevines [40]. In this case, RNA-Seq data showed significant transcriptional upregulation of genes involved in PA synthesis following SA treatment (Fig. 3c). In particular, *FaUFGTs* were downregulated in the SA treatment (Fig. 3c), indicating that SA could induce and increase PAs accumulation. The MBW complex (MYB–bHLH–WD40) regulates the biosynthesis of PAs via directly activation of the genes involved in the late steps of the flavonoid biosynthetic pathway [41]. It is generally known that the function of PAs from different tissues (fruits, leaves, and stems) is to protect against pathogens, insects, and herbivores [40]. In an effort to broaden our knowledge of MBW-mediated PAs accumulation in strawberry leaf, phylogenetic analysis was made with previous MYB member proteins from strawberry fruit (Fig. 4). The phylogenetic analysis showed that *FaMYB9*, *FaMYB11*, *FabHLH3*, and *FaTTG1* may be functional component in the formation of the MBW complex involved in PA biosynthesis (Fig. 4 abc). An increase in *FaMYB9*, *FaMYB11*, *FabHLH3*, and *FaTTG1* expression levels was observed in the SA treatment, which coincided with higher PAs content (Fig. 3a) and expression levels of key structural genes (Fig. 3c). In strawberry leaves, in particular, a relatively high concentration of PAs was observed at 3 dpi under SA treatment (Fig. 1 Ac). These transcriptional modifications were in agreement with the MBW complex was shown to regulate the PA biosynthesis [28]. Our results favor a scenario that FabHLH3 may be more related to PAs biosynthesis, in association with FaMYB9 and FaMYB11, forming a ternary complex (FaMYB9/FaMYB11, FabHLH3, and FaTTG1), leading to upregulate PAs content against *P. aphains* in strawberry leaves. In strawberry fruit, FaMYB5 could interact with FaMYB1 to regulate the MBW complexes [28, 41]. In the case of three *FaMYB5* genes, upregulation was observed in SAInfected, indicating that there is a correlation between SA signaling pathway and *FaMYB5* expression. It is interesting that SA could induce PAs accumulation, suggesting that the mechanism that regulated PAs accumulation might act dependently from SA- or JA-signaling pathways. The possibility that *FaMYB5* may promote PAs biosynthesis in strawberry leaves. Therefore, *FaMYB5* may have a role in fine-tuning PA biosynthesis during strawberry leaves response to *P. aphanis*. It is noteworthy that PAs is antifungal metabolites against *P. aphanis*.

### DEGs involved in SA signaling pathway result to defence against *P. aphanis*

It is known that the SA-mediated pathway elevates resistance to biotrophs. NPR1 is a key positive regulator of SA signalling transduction and physically interacts with TGA, which is involved in SA-dependent activation of *PR1*, leading to transcriptional regulation of gene defence systems [9, 42]. *FaNPR3-like* was phylogenetically closer to *FvNPRL-1* (Fig. 5a), which may be repressor of SA-signalling pathway. Furthermore, *FaTGA* (similar to *FaTGA1* and *AtTGA1*) likely function as positive regulators of SA biosynthesis and SA signalling pathway. The WRKY family of genes mediates plant defences against various pathogens [43]. WRKY70 is an important molecule involved in the balance between SA- and JA-dependent responses [44]. WRKY70 positively regulates SA-mediated signalling by increasing expression of EDS1 in *Arabidopsis* [11]. A strong expression of *FaWRKY70* (similar to *SlWRKY70* and *AtWRKY70*) was induced by SA treatment, suggesting that *FaWRKY70* play a crucial role in SA signalling pathway. Moreover, an obvious resistance against *P. aphains* (Figs. 1b and 2) in combination with a strong induction of *FaWRKY70* (Fig. 5) suggest a regulator of downstream responses mediated by SA-JA signaling cross talk, is involved in the positive regulation of *P. aphains*. Application of SA results to the rapid induction of *FaNPR3-like*, which may remove excessive SA. As NPR1 paralogs, NPR3 and NPR4 were regulators in both SA signaling pathway [45] and JA signaling pathway [46]. However, exogenous SA induced a strong upregualtion of *FaPR1* (Fig. 6). It clear that NPR1 interacts with TGA, which are necessary for *PR1*. In our study, NPR1 homologs have not been identified.

### DEGs involved in JA biosynthesis and signaling pathway response to *P. aphanis* infection

Emerging evidence has demonstrated SA–JA antagonism in many plants [27, 47, 48]. SA levels need to be regulated to defense against pathogen, followed by a reduction in expression patterns of JA-biosynthesis genes, suggesting strawberry activate SA signaling pathway and suppress JA signaling pathway. JAZ plays a crucial role in repressing JA responses, in which the function of MYC2 is repressed by JAZ [49]. *FaJAZ* showed a reduction pattern from 0 dpi to 3 dpi in two groups, accompanied by significant expression levels of *FaJAZ* induced by SA, indicating that *P. aphinas* might inhibit the expression levels of *FaJAZ*. MYC2 is a direct target for JAZ in the JA signalling pathway [50]. *FaJAZ10–1* and *FaJAZ1–2* exhibited similar upregulation pattern to *FaMYC2*, while *FaJAZ1* (Fig. 4b) and *FaMYC2-like* (Fig. 4d) showed strong induction, which are JA-responsive genes and related with the activation of JA biosynthesis genes induced by exogenous SA, indicating that SA-treated leaves elevated JA biosynthesis in the early stage, which is in agreement with that the MeJA-induce anthocyanin accumulation in strawberry fruit [39]. Moreover, SA levels during *P. aphains* infection correlates with the accumulation of PAs in strawberry leaves, indicating that exogenous SA could induce accumulation of PAs to defense against *P. aphanis*. Therefore, the SA pathway could act antagonistically with JA for PAs accumulation in strawberry leaves response to *P. aphains*.

The induction of SA caused by *P. aphanis* attack, or an exogenous application of SA can facilitate stronger responses to *P. aphanis* [33]. Indeed, no significant difference in the transcript expression of *FaJAR1*, *FaLOX2*, and *FaAOS* between 3dpi/0dpi in two groups was observed (Figure S6). Moreover, *FaJAR1*, *FaLOX2*, and *FaAOS* levels after 4 h of exogenous SA were higher than in ddH_2_O pretreatment. The data presented here showed that exposure of strawberry to SA is sufficient to induce an early enhanced levels of JA biosynthesis genes, which is associated with that exogenous SA can induced JA biosynthesis, leading to accumulation of PAs. Together, these results also support the notion that application of SA has the ability to activation of SA signalling pathway and override JA signaling pathway. Supporting this concept, the *FaPAD4* and *FaEDS1* transcript did increase after JA treatment in ‘Camarosa’ [51].

### Defence-related proteins contribute to enhanced resistance to *P. aphanis*

In accordance with the results of the resistance performance assays (Fig. 1), nine DEGs encoding PR proteins identified by transcriptome data (log2 fold change > 3) were found to be linked with increased resistance to *P. aphanis*, including PR1, PR2, PR3, PR5, PR9, and PR10. Compared with ddH_2_O-treated group, these nine PR genes were specifically resistant to *P. aphanis* or expressed at higher levels in SA-treated group (Fig. 5, Tables S4). In particular, *PR1*, *PR2–1*, *PR2–2*, *PR3*, *PR5–1*, and *PR5–2* showed higher expression in SA treatment than in ddH_2_O treatment across the experimental time, especially significant upregulation at 1 dpi prior to obvious white symptom (Fig. 2b), highlighting that these PR genes are consequences of SA signalling in resistance to *P. aphanis*. Therefore, it is likely that PR1, PR2, PR3, and PR5 might also be an antifungal metabolite against *P. aphanis*.

## Conclusions

**Fig. 7:**
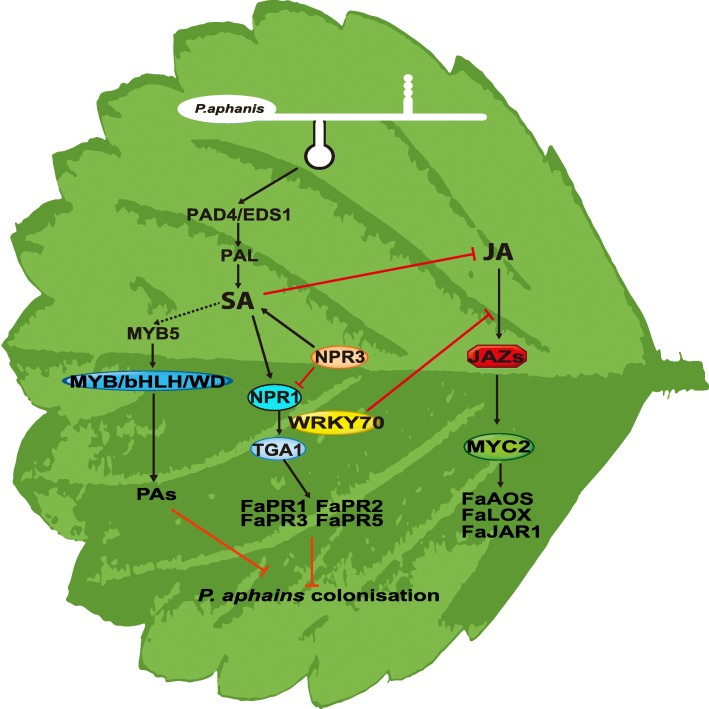
Model depicting the potential regulatory mechanisms involved in resistance to *P. aphanis* in strawberry leaves primed by SA. In SA-pretreated plants, several genes related to resistance to *P. aphanis* were activated: genes involved in flavonoid biosynthesis, SA biosynthesis, and SA signal transduction. SA induced strawberry the expression of PR genes (*PR1*, *PR2*, *PR3*, and *PR5*). Among the transcription factors involved in the plant hormone signal transduction pathways, *FaTGA*, F*aWRKY70*, and *FaMYC2* were highlighted as possible regulators of the network of SA-induced defence. PAs acts in an antifungal manner and accumulate through stimulation of their biosynthesis genes, suggesting a potential role in defence against *P. aphains*

We identified different candidate genes in the octoploid strawberry response to *P. aphanis* compared to those identified in the study on PR genes and TFs in the diploid strawberry–*P. aphanis* interaction. Similar to the binary Arabidopsis model with SA-JA crosstalk model in *Arabidopsis* [52], an integrated model of the defence response to *P. aphanis* in strawberry was proposed (Fig. 7). We suggest the following conclusions: (i) *P. aphanis* induces drastic changes in gene expression and metabolite production in strawberry; (ii) SA primes the strawberry with enhanced resistance, providing a constraint of SA-induced resistance against *P. aphanis*; (iii) unlike *Arabidopsis thaliana*, the SA biosynthetic pathway of strawberry may be mainly derived from the PAL pathway; (iv) application of exogenous SA could induce the accumulation of PAs by activation of MBW complexes; (v) upregulation of PR genes are observed during the resistance response to *P. aphanis*, in particularly *PR1*, *PR2–1*, *PR2–2*, *PR3*, *PR5–1*, and *PR5–2*; (vi) several TFs involved in phytohormone signalling pathway contributes to resistance to *P. aphanis*. Comparative transcriptome analysis enabled us to uncover a novel resistance mechanism associated with SA signalling, followed by significant resistance against *P. aphanis*. This study lays the foundation for further exploration of the molecular mechanisms of resistance to *P. aphanis* in strawberry, and provides new strategies for improving strawberry varieties through genetic engineering.

## Methods

### Plant materials

Seedlings of strawberry (*Fragaria × ananassa* Duch.) ‘Benihoppe’ were provided by the National Germplasm Repository of Strawberry, Beijing Academy of Agriculture and Forestry Sciences, China. The experimental plants were propagated from runners, which were rooted in 5-cm diameter pots filled with a 1:1:1 mixture of peat: vermiculite: perlite and subsequently transplanted in a greenhouse on September 15, 2018 into 20-cm diameter pots filled with a 1:1:1 mixture of peat: vermiculite: perlite. The inoculation experiment was conducted in a plastic greenhouse at Beijing Forestry University. The growth conditions during the experiment period are shown in Figure S1. No fungicides were applied, and fertiliser was added according to agricultural practice.

### *P. aphanis* inoculation and SA treatment

*P. aphanis* inoculation was performed one month later on October 15, 2018. The strawberry leaf was infected with *P. aphanis* by gentle tapping from an infected strawberry leaf. The young and healthy plants inoculated with *P. aphanis* replaced infected old plants. The inoculation experiment was conducted by shaking down gentle from above leaves infected by powdery mildew to locate on the adaxial epidermis of 14 day-old full-extending young leaves of the strawberry. The amount of conidia counted to approximately 200–300 spores per cm^2^. All experimental groups were placed in a greenhouse under the growth conditions described above. Eighty plants were split into two subgroups containing 40 plants each: ddH_2_O-treated group and SA-treated group. Four hours before inoculation with *P. aphanis*, one group was sprayed with water, and at the same time another group was sprayed with exogenous SA (2 mM) using an atomizer onto the upper fully leaves until it ran off. Three replicates were sampled for each infection time. Randomly ten upper young leaves from each plant were sampled at 0, 1, 3, 5, and 7 dpi and RNA was extracted. All collected leaves were inoculated by *P. aphains* and marked with label, while inoculated area is 16.73 to 25.37% of the entire leaf area. Samples were immersed in liquid nitrogen and kept at − 80 °C until further analysis. This study evaluated four conditions: water treatment and no inoculation (ddH_2_OUninfected); SA treatment and no inoculation (SAUninfected); water treatment with inoculation at 3 dpi (ddH_2_OInfected); and SA treatment with inoculation at 3 dpi (SAInfected).

### TFC and PAs determination

Total flavonoid content (TFC) was determined by a colorimetric method, according to plant total phenol test kit (A142–1-1, Nanjing Jiancheng Bioengineering Institute, Nanjing, China). Briefly, sodium nitrite was added to sample, and then aluminum chloride was added. Finally, odium hydroxide was added to the mixture. After 2 h, the sample extract was centrifuged for 10 min at 10,000 rpm, and the absorbance of the supernatant was read at 502 nm and compared with that of rutin standards. The flavonoid content is expressed as mg g^− 1^ DW (dry weight).

Proanthocyanidins content (PAs) was determined according to plant proanthocyanidins test kit (A144–1-1, Nanjing Jiancheng Bioengineering Institute, Nanjing, China). The chromogenic solution, vanillin solution: hydrochloric acid solution = 1:1, ready-to-use. Briefly, methanol was added to sample. After 20 min stirring, the mixture was centrifuged for 20 min at 3000 rpm, and then supernatant was added to the chromogenic solution. The absorbance of the solution was measured at 500 nm and compared with that of rutin standards. The PAs content is expressed as mg g^− 1^ FW (fresh weight).

### Extraction of endogenous free SA

Phytohormones were analyzed according to the standard protocol described by Pan et al. [28]. Triplicate samples of 50 mg of leaves were used, and liquid chromatography-mass spectrometry (Waters Crop., Milforf, MA) analyses of SA were performed by the Testing and Analysis Center of Beijing Forestry University, Beijing, China.

### Assays of fluorescence parameters

The Imaging-PAM Chlorophyll Fluorometer (Walz, Effeltrich, Germany) was used to determine photosynthetic parameters of infected strawberry leaves [32]. Plantlets were prepared with a 20 min dark treatment. Using the Imaging-PAM, Chlorophyll Fluorometer, dark-treatment fluorescence yield, *F*_*0*_, and the maximum fluorescence yield, *F*_*m*_, were determined. Walz software used the formula *F*_*v*_*/F*_*m*_ = (*F*_*m*_-*F*_*0*_)/*F*_*m*_ to calculate the maximal PSII quantum yield. The current fluorescence yield, *F*_*t*_, and the maximum light-adapted fluorescence, *F*_*m′*_, in the presence of actinic illumination of 400 μmol m^− 2^ s^− 1^ were used to compute the effective PSII quantum yield [Y (II) = (*F*_*m′*_–*F*_*t*_)/*F*_*m′*_]. NPQ was determined as the quotient (*F*_*m*_-*F*_*m′*_)/*F*_*m*_. The non-regulated energy dissipation of PSII quantum field Y (NO) was obtained using the following formula: Y (NO) = 1/(NPQ + 1 + qL (*F*_*m*_/*F*_*0*_–1)). Following the kinetics recording, Walz software used areas of interest that were randomly selected from corresponding surrounding areas of infected leaves. For each treatment, three plants were used and the values were averaged.

### Light microscopy

Light microscopy analyses of the development of *P. aphanis* [33] were performed on leaf discs (20 mm diameter) randomly excised from infected leaves at 0, 1, 3, 5, 7 dpi. Leaf disc were stained by boiling for 2 min in alcoholic lactophenol trypan blue (10 mL ethanol, 10 mL phenol, 10 mL water, 10 mL lactic acid, and 10 mg trypan blue). The stained discs were cleared in chloral hydrate (2.5 g dissolved in 1 mL of water) overnight at room temperature. Cleared leaves were mounted under coverslips in 50% glycerol and observed using a Leica DM2500. *P. aphanis* was examined using confocal laser scanning microscopy (Heidelberg Engineering GmbH, Germany).

### Transcriptome analysis

To identify the key pathways in the strawberry–*P. aphanis* interaction, twelve samples (ddH_2_OUninfected, ddH_2_OInfected, SAUninfected, and SAInfected; three replicates per treatment) were used for RNA-Seq analysis (Table S1). Total RNA was extracted and an RNA-Seq transcriptome library was prepared using the TruSeqTM RNA Sample Preparation Kit (Illumina, San Diego, CA, USA). Sequence reads were aligned using the sequence assembly of the genome annotation (Fragaria_x_ananassa_Camarosa_Genome_v1.0.a1) as a reference, which is available at Genome Database for Rosaceae (GDR, ftp://ftp.bioinfo.wsu.edu/species/Fragaria_x_ananassa/). To identify DEGs (differential expression genes) between two different samples, the expression level of each transcript was calculated according to the Fragments Per Kilobase Million (FPKM) method. RSEM (http://deweylab.biostat.wisc.edu/rsem/) was used to quantify gene abundances. R statistical package software DESeq2 (http://bioconductor.org/packages/stats/bioc/DESeq2/) was utilized for differential expression analysis.

### Phylogenetic tree and protein motifs analysis

The gene IDs of the genes from the strawberry are shown in Tables S2 and S3. The full predicted amino acid sequences of the genes from the two plant species (strawberry and Arabidopsis) were aligned using ClustalW (opening = 10, extension = 0.2). Phylogeny reconstruction was done using the neighbour-joining method and tested using the bootstrap method with 1000 replicates. Both alignment and phylogenetic analysis were performed using MEGA version 7.0 software. The scale bar indicates the branch length that corresponds to the number of substitutions per amino acid position. The MEME program (http://meme.nbcr.net/meme3/mme.html) was used to predict the motifs of all proteins, which were further annotated by the motiffinder (https://www.genome.jp/tools/motif/).

### Validation of RNA-Seq data by RT–qPCR

Ten transcriptomic genes from the RNA-Seq analysis were selected and validated by RT-qPCR. Primers were designed using Primer 5.0 (Table S6). Ten selected key homologs were *PR1* (*FxaC_7g01820*), *PR2* (*FxaC_9g22040* and *FxaC_21g36890*), *PR3* (*FxaC_3g11800*), *PR5* (*FxaC_21g46690* and *FxaC_24g22200*), *PR9* (*FxaC_12g01950*), *PR10* (*FxaC_14g19400*), *PAD4* (*Fxa_C2g34100*) and *EDS1* (*Fxa_C17g21160*). Total RNA from leaves was isolated from each sample using EASYspin and the plant RNA Mini Kit (Aidlab, Beijing, China). Subsequently, RNase-free DNase was used to treat total RNA (2 μg) to remove genomic DNA. cDNA was synthesized using GoScript Reverse Transcription Kit according to the manufacturer’s instructions (CWBIO, Jiangsu, China). RT-qPCR was performed in 20 μl reactions using an Applied Biosystem 7500 real-time PCR system. Gene expression was calculated using the 2^−ΔΔCt^ method [53] and normalised using two *FaACTIN* genes as internal control. Reactions were performed with three biological replicates.

### Statistical analysis

Experiment was carried out following a completely randomized design (three replications). Data were expressed as the means ± SD (*n* = 3), and analysed using ANOVA by the SPSS 17.0 (SPSS Inc., USA). The level of significance difference was established at *P* < 0.05. For RNA-seq, *P*-values were adjusted using q-values, q-value< 0.005 and |log2(foldchange)| ≥ 1 were set as the threshold for significantly differential expression. PCA were made with ggplot2. Differences were analysed using a one-way analysis of variance with Fisher’s least significant difference test. P-value ≤0.05 were considered statistically significant. All analyses were performed using Origin 8.0 software (OriginLab Corp., https://www.originlab.com/).

## Supplementary information

**Additional file 1 **: **Figure S1**. Temperature (red) and humidity (green) in the greenhouse during the experimental period. The data were collected every 2 h from October 15 to October 22, 2018.

**Additional file 2 **: **Figure S2**. The principle component analysis (PCA) of differently expressed transcripts during strawberry-*P. aphanis* interaction. PC1 separates samples according to genotype and explains 50.96% of variance. PC2 separates samples according to the different infection time in that plants were exposed to *P. aphanis* colonisation and explains 13.44% of the variance. Orange, purple, green, and blue represents ddH_2_OUninfected, ddH_2_OInfected, SAUninfected, and SAInfected.

**Additional file 3 **: **Figure S3**. DEGs in ddH_2_O-treated and SA-treated leaves. (a) The number of DEGs up- or downregulated at 3 dpi in both groups. (b) Heatmap generated from the DEGs from RNA-Seq analysis comparing the fold changes in gene expression during the infection stage. Heatmap colours represent gene expression fold-change levels based on the provided colour key scale; red = upregulated expression, blue = downregulated expression, and white = no change in expression. (c) Venn diagrams of the number of DEGs between ddH_2_O-treated and SA-treated groups. (d) Venn diagram showing a cross-comparison of the up- and downregulated genes from both groups. (e) Cluster analysis of DEGs in the ddH_2_O-treated and SA-treated groups based on the BIRCH method.

**Additional file 4 **: **Figure S4**. (a) Enriched Gene Ontology (GO) biological processes in ddH_2_OInfected/ddH_2_OUninfected. (b) Enriched Gene Ontology (GO) biological processes in SAInfected/SAUninfected. The y-axis represents enriched GO processes (false discovery rate < 0.05). The x-axis indicates the total number of genes annotated to each GO process. Red and blue sections represent downregulated and upregulated genes, respectively. (c) KEGG enrichment pathways (Top 20) for ddH_2_OInfected vs ddH_2_OUninfected. (d) KEGG enrichment pathways (Top 20) for SAInfected vs SAUninfected. The rich factor indicates the degree of enrichment. The colour and size of the dots indicate the range of the q-value and gene number, respectively.

**Additional file 5 **: **Figure S5**. Hierarchical clustering heatmap for DEGs in the ddH_2_OInfected/ddH_2_OUninfected and SAInfected/SAUninfected. (a) Cluster analysis of DEGs involved in plant hormone signalling between the ddH_2_OInfected/ddH_2_OUnifected; (b) cluster analysis of DEGs involved in plant hormone signalling between the SAInfected/SAUninfected. Differences are highlighted in blue (downregulation) and red (upregulation).

**Additional file 6 **: **Figure S6**. Identification of putative genes involved in SA biosynthesis and JA biosynthesis in strawberry leaves infected by *P. aphanis*. The phylogenetic relationships existing between the strawberry PAL plus ICS (a), EDS1 plus PAD4 (b), JAR1 (c), AOS1 (d), and LOX (e) proteins used in this study (black arrow) with their closest homologues. Numbers at branches indicate posterior probabilities and bootstrap percentages based on 1000 replicates. (f) Changes in relative expression of *FaPAL*, *FaICS2*, *FaEDS1*, *FaPAD4*, *FaJAR*, *FaAOS* and *FaLOX* genes at 0 dpi and 3 dpi in both groups. The values were normalized against controls and correspond to the mean of three biological replicates ± S.E. Asterisks indicate significant differences (* *p* < 0.05). PAL, phenylalanine ammonia-lyase; ICS, isochorismate Synthase; EDS2, enhanced disease susceptibility2; PAD4, phytoalexin deficient 4; JAR1, JA-amino acid synthetase1; AOS, allene oxide synthase; LOX, lipoxygenase.

**Additional file 7 **: **Table S1**. RNA-Seq Reads and Reads Mapping. **Table S2**. Differentially expressed genes in 3DPI−/Control- (|log2FC| > 1). **Table S3**. Differentially expressed genes in SAInfected/SAUninfected (|log2FC| > 1). **Table S4.** Significant DEGs involved in flavonoid pathway between SAInfected/SAUninfected. **Table S5**. Significant DEGs (log2 fold change > 3) in SAInfected/SAUninfected. **Table S6**. Primer sequences and parameters derived from RT-qPCR analysis.

## Data Availability

All data sustaining the results in this study are included in this article or its supplementary information files. The raw sequence data reported in this paper have been deposited in the Genome Sequence Archive [47] in BIG Data Center [48], Beijing Institute of Genomics (BIG), Chinese Academy of Sciences, under accession numbers CRA001964, which are publicly accessible at https://bigd.big.ac.cn/gsa.

## Abbreviations

BTH: Benzothiadiazole
DEG: Differentially expressed gene
FPKM: Fragments per kilobase of transcript per million
*F*_*v*_*/F*_*m*_: Photosynthetic efficiency
GO: Gene ontology
HR: Hypersensitive response
KEGG: Kyoto encyclopedia of genes and genomes
*Φ*_*NPQ*_: Nonphotochemical quenching
PCA: Principal component analysis
PR: Pathogensis-related genes
RT-qPCR: Real-time quantitative polymerase chain reaction
RNA-Seq: RNA-sequencing
ROS: Reactive oxygen species
SA: Salicylic acid
TFC: Total flavone content
TFs: Transcription factors
*Φ*_*PSII*_: The actual PSII efficiency
*Φ*_*NO*_: Quantum yield of unregulated energy dissipation

## References

[1] Jones JD, Dangl JL (2006). The plant immune system. Nature..

[2] Chisholm ST, Coaker G, Day B, Staskawicz BJ (2006). Host-microbe interactions: shaping the evolution of the plant immune response. Cell..

[3] Braun U, Cook R, Inman A, Shin H (2002). The taxonomy of the powdery mildew fungi. The powdery mildews: a comprehensive treatise.

[4] Takamatsu S (2013). Molecular phylogeny reveals phenotypic evolution of powdery mildews (*Erysiphales, Ascomycota*). J Gen Plant Pathol.

[5] Glawe DA (2008). The powdery mildews: a review of the world's most familiar (yet poorly known) plant pathogens. Annu Rev Phytopathol.

[6] Kuhn H, Kwaaitaal M, Kusch S, Acevedo-Garcia J, Wu H, Panstruga R (2016). Biotrophy at its best: novel findings and unsolved mysteries of the Arabidopsis-powdery mildew pathosystem. Arabidopsis Book.

[7] Robert-Seilaniantz A, Grant M, Jones JD (2011). Hormone crosstalk in plant disease and defense: more than just jasmonate-salicylate antagonism. Annu Rev Phytopathol.

[8] Spoel SH, Koornneef A, Claessens SM, Korzelius JP, Van Pelt JA, Mueller MJ, Buchala AJ, Métraux J-P, Brown R, Kazan K (2003). NPR1 modulates cross-talk between salicylate-and jasmonate-dependent defense pathways through a novel function in the cytosol. Plant Cell.

[9] Johnson C, Boden E, Arias J (2003). Salicylic acid and NPR1 induce the recruitment of trans-activating TGA factors to a defense gene promoter in Arabidopsis. Plant Cell.

[10] Zander M, Chen S, Imkampe J, Thurow C, Gatz C (2012). Repression of the Arabidopsis thaliana jasmonic acid/ethylene-induced defense pathway by TGA-interacting glutaredoxins depends on their C-terminal ALWL motif. Mol Plant.

[11] Li J, Brader G, Palva ET (2004). The WRKY70 transcription factor: a node of convergence for jasmonate-mediated and salicylate-mediated signals in plant defense. Plant Cell.

[12] Spoel SH, Dong X (2008). Making sense of hormone crosstalk during plant immune responses. Cell Host Microbe.

[13] Thaler JS, Humphrey PT, Whiteman NK (2012). Evolution of jasmonate and salicylate signal crosstalk. Trends Plant Sci.

[14] Folta KM, Gardiner SE (2009). Genetics and genomics of Rosaceae.

[15] Ainsworth GC (2008). Ainsworth & Bisby's dictionary of the fungi.

[16] Zhang Y, Wang G, Dong J, Zhong C, Chang L, Zhang H (2016). The current progress in strawberry breeding in China. VIII International Strawberry Symposium 1156.

[17] Edger PP, Poorten TJ, VanBuren R, Hardigan MA, Colle M, McKain MR, Smith RD, Teresi SJ, Nelson AD, Wai CM (2019). Origin and evolution of the octoploid strawberry genome. Nat Genet.

[18] Jambagi S, Dunwell JM. Global transcriptome analysis and identification of differentially expressed genes after infection of *Fragaria vesca* with powdery mildew (Podosphaera aphanis). Transcriptomics: Open Access. 2015;3(1).

[19] Hu Y, Han Y, Wei W, Li Y, Zhang K, Gao Y, Zhao F, Feng J (2015). Identification, isolation, and expression analysis of heat shock transcription factors in the diploid woodland strawberry Fragaria vesca. Front Plant Sci.

[20] Jambagi S, Dunwell JM (2017). Identification and expression analysis of Fragaria vesca MLO genes involved in interaction with powdery mildew (*Podosphaera aphanis*). J Adv Plant Biol.

[21] Wei W, Cui M-Y, Hu Y, Gao K, Xie Y-G, Jiang Y, Feng J-Y (2018). Ectopic expression of FvWRKY42, a WRKY transcription factor from the diploid woodland strawberry (Fragaria vesca), enhances resistance to powdery mildew, improves osmotic stress resistance, and increases abscisic acid sensitivity in Arabidopsis. Plant Sci.

[22] Jiwan D, Roalson EH, Main D, Dhingra A (2013). Antisense expression of peach mildew resistance locus O (*PpMlo1*) gene confers cross-species resistance to powdery mildew in Fragaria x ananassa. Transgenic Res.

[23] Silva KJP, Brunings A, Peres NA, Mou Z, Folta KM (2015). The Arabidopsis NPR1 gene confers broad-spectrum disease resistance in strawberry. Transgenic Res.

[24] Silva KJP, Brunings AM, Pereira JA, Peres NA, Folta KM, Mou Z (2017). The Arabidopsis *ELP3/ELO3* and *ELP4/ELO1* genes enhance disease resistance in Fragaria vesca L. BMC Plant Biol.

[25] Cockerton HM, Vickerstaff RJ, Karlström A, Wilson F, Sobczyk M, He JQ, Sargent DJ, Passey AJ, McLeary KJ, Pakozdi K (2018). Identification of powdery mildew resistance QTL in strawberry (Fragaria× ananassa). Theor Appl Genet.

[26] Pertot I, Zasso R, Amsalem L, Baldessari M, Angeli G, Elad Y (2008). Integrating biocontrol agents in strawberry powdery mildew control strategies in high tunnel growing systems. Crop Prot.

[27] Dodgson JLA (2007). Epidemiology and sustainable control of Podosphaera aphanis (strawberry powdery mildew).

[28] Schaart JG, Dubos C, Romero De La Fuente I, Van Houwelingen AM, de Vos RC, Jonker HH, Xu W, Routaboul JM, Lepiniec L, Bovy AG (2013). Identification and characterization of MYB-bHLH-WD40 regulatory complexes controlling proanthocyanidin biosynthesis in strawberry (F ragaria× ananassa) fruits. New Phytol.

[29] Sun T, Busta L, Zhang Q, Ding P, Jetter R, Zhang Y (2017). TGACG-BINDING FACTOR 1 (TGA1) and TGA4 regulate salicylic acid and pipecolic acid biosynthesis by modulating the expression of SYSTEMIC ACQUIRED RESISTANCE DEFICIENT 1 (SARD1) and CALMODULIN-BINDING PROTEIN 60g (CBP60g). New Phytol.

[30] Li J (2004). The WRKY70 transcription factor : a node of convergence for jasmonate-mediated and salicylate-mediated signals in plant defense. Plant Cell.

[31] Atamian HS, Eulgem T, Kaloshian I (2012). SlWRKY70 is required for Mi-1-mediated resistance to aphids and nematodes in tomato. Planta.

[32] Lin-Jie S, Jui-Yu L, Nai-Chun L, Chia-Lin C (2018). Identification of a strawberry NPR-like gene involved in negative regulation of the salicylic acid-mediated defense pathway. PLoS One.

[33] Feng J, Cheng Y, Zheng C (2019). Expression patterns of octoploid strawberry TGA genes reveal a potential role in response to Podosphaera aphanis infection. Plant Biotechnol Rep.

[34] Chen C, Chen Z (2000). Isolation and characterization of two pathogen- and salicylic acid-induced genes encoding WRKY DNA-binding proteins from tobacco. Plant Mol Biol.

[35] Wei J, van Loon JJA, Gols R, Menzel TR, Li N, Kang L, Dicke M (2014). Reciprocal crosstalk between jasmonate and salicylate defence-signalling pathways modulates plant volatile emission and herbivore host-selection behaviour. J Exp Bot.

[36] Chini A, Gimenez-Ibanez S, Goossens A, Solano R (2016). Redundancy and specificity in jasmonate signalling. Curr Opin Plant Biol.

[37] Garrido-Bigotes A, Figueroa NE, Figueroa PM, Figueroa CR. Jasmonate signalling pathway in strawberry: Genome-wide identification, molecular characterization and expression of JAZs and MYCs during fruit development and ripening. PloS one. 2018;13(5):e0197118. 10.1371/journal.pone.0197118 PMC5944998 29746533

[38] Preuss A, Augustin C, Hoffmann T, Figueroa CR, Valpuesta V, Sevilla JF, Schwab W (2014). Expression of a functional jasmonic acid carboxyl methyltransferase is negatively correlated with strawberry fruit development. J Plant Physiol.

[39] Garrido-Bigotes A, Figueroa PM, Figueroa CR (2018). Jasmonate metabolism and its relationship with Abscisic acid during strawberry fruit development and ripening. J Plant Growth Regul.

[40] Dixon RA, Xie D-Y, Sharma SB (2005). Proanthocyanidins – a final frontier in flavonoid research?. New Phytol.

[41] Aharoni A, Vos CHRD, Wein M, Sun Z, O'Connell AP (2001). The strawberry FaMYB1 transcription factor suppresses anthocyanin and flavonol accumulation in transgenic tobacco. Plant J.

[42] Despres C (2003). The Arabidopsis NPR1 disease resistance protein is a novel cofactor that confers redox regulation of DNA binding activity to the basic domain/leucine zipper transcription factor TGA1. Plant Cell.

[43] Pandey SP, Somssich IE (2009). The role of WRKY transcription factors in plant immunity. Plant Physiol.

[44] Li J, Brader G, Kariola T, Tapio Palva E (2006). WRKY70 modulates the selection of signaling pathways in plant defense. Plant J.

[45] Zhang Y, Cheng YT, Qu N, Zhao Q, Bi D, Li X (2006). Negative regulation of defense responses in Arabidopsis by two NPR1 paralogs. Plant J.

[46] Liu L, Sonbol F-M, Huot B, Gu Y, Withers J, Mwimba M, Yao J, He SY, Dong X (2016). Salicylic acid receptors activate jasmonic acid signalling through a non-canonical pathway to promote effector-triggered immunity. Nat Commun.

[47] Wang Y, Song F, Zhu J, Zhang S, Yang Y, Chen T, Tang B, Dong L, Ding N, Zhang Q (2017). GSA: genome sequence archive. Genomics Proteomics Bioinformatics.

[48] Zhang Z, Zhao W, Xiao J, Bao Y, He S, Zhang G, Li Y, Zhao G, Chen R, Gao Y, Zhang C (2020). Database resources of the national genomics data center in 2020. Nucleic Acids Res.

[49] Melotto M, Mecey C, Niu Y, Chung HS, Katsir L, Yao J, Zeng W, Thines B, Staswick P, Browse J (2008). A critical role of two positively charged amino acids in the Jas motif of Arabidopsis JAZ proteins in mediating coronatine-and jasmonoyl isoleucine-dependent interactions with the COI1 F-box protein. Plant J.

[50] Zhai Q, Yan L, Tan D, Chen R, Sun J, Gao L, Dong M-Q, Wang Y, Li C (2013). Phosphorylation-coupled proteolysis of the transcription factor MYC2 is important for jasmonate-signaled plant immunity. PLoS Genet.

[51] Amil-Ruiz F, Garrido-Gala J, Gadea J, Blanco-Portales R, Muñoz-Mérida A, Trelles O, de Los Santos B, Arroyo FT, Aguado-Puig A, Romero F, Mercado JÁ, Pliego-Alfaro F, Muñoz-Blanco J, Caballero JL. Partial Activation of SA- and JA-Defensive Pathways in Strawberry upon Colletotrichum acutatum Interaction. Front Plant Sci. 2016;7:1036. https://www.ncbi.nlm.nih.gov/pmc/articles/PMC4945649/. 10.3389/fpls.2016.01036 PMC4945649 27471515

[52] Spoel SH, Dong X (2012). How do plants achieve immunity? Defence without specialized immune cells. Nat Rev Immunol.

[53] Livak K, Schmittgen T (2001). Analysis of relative gene expression data using real-time quantitative PCR and the ^2-△△Ct^ method. Methods..

