# Supplementary figures and images for: Salicylic acid-primed defence response in octoploid strawberry ‘Benihoppe’ leaves induces resistance against Podosphaera aphanis through enhanced accumulation of proanthocyanidins and upregulation of pathogenesis-related genes
Source: BMC Plant Biol. 2020 Apr 8;20:149. doi: 10.1186/s12870-020-02353-z (PMC7140339; doi:10.1186/s12870-020-02353-z)

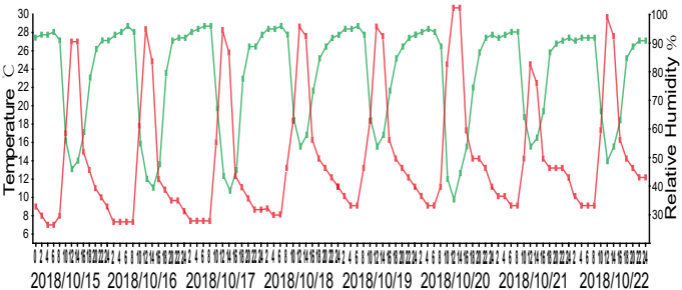

Supplement: Supplementary file 1 — Additional file 1 : Figure S1. Temperature (red) and humidity (green) in the greenhouse during the experimental period. The data were collected every 2 h from October 15 to October 22, 2018. [file 12870_2020_2353_MOESM1_ESM.pdf]

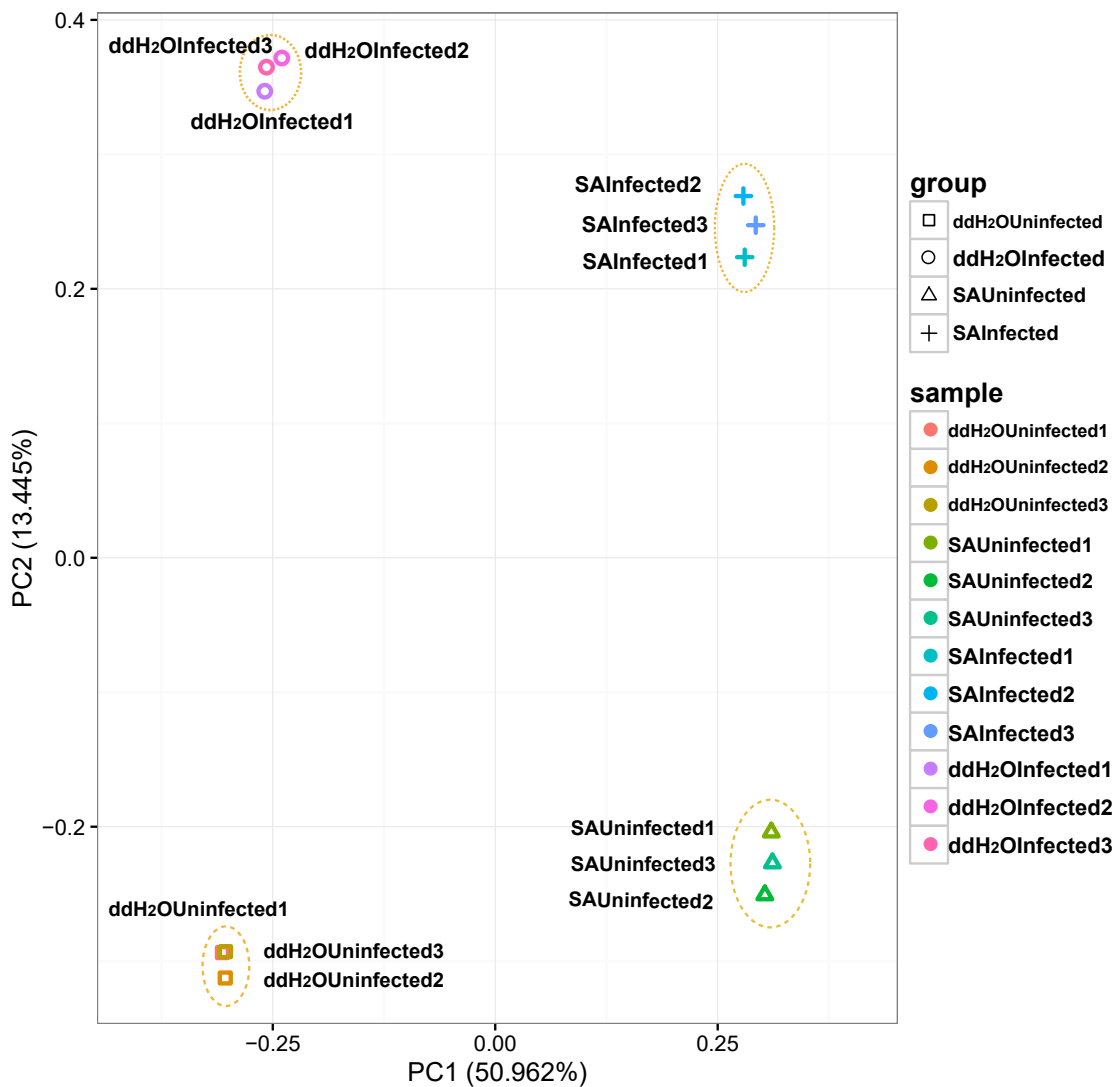

Supplement: Supplementary file 2 — Additional file 2 : Figure S2. The principle component analysis (PCA) of differently expressed transcripts during strawberry-P. aphanis interaction. PC1 separates samples according to genotype and explains 50.96% of variance. PC2 separates samples according to the different infection time in that plants were exposed to P. aphanis colonisation and explains 13.44% of the variance. Orange, purple, green, and blue represents ddH2OUninfected, ddH2OInfected, SAUninfected, and SAInfected. [file 12870_2020_2353_MOESM2_ESM.pdf]

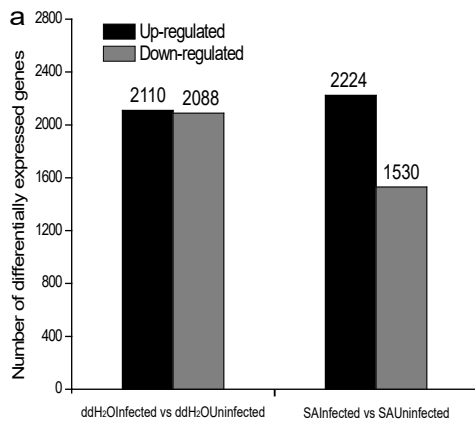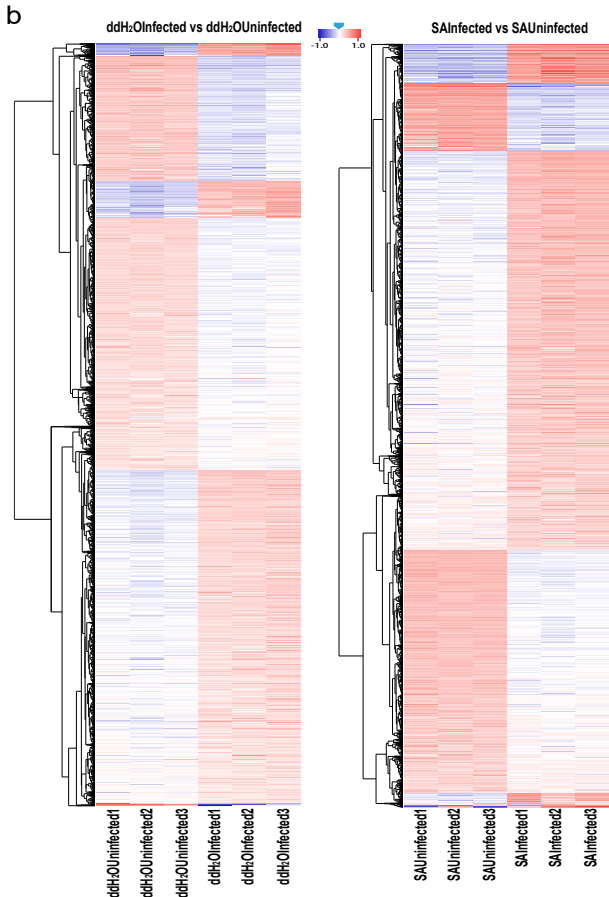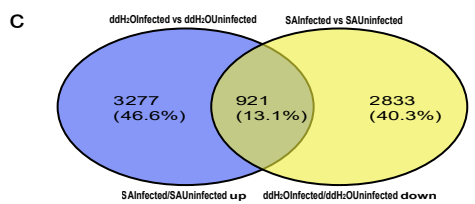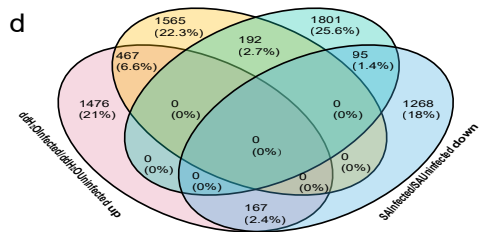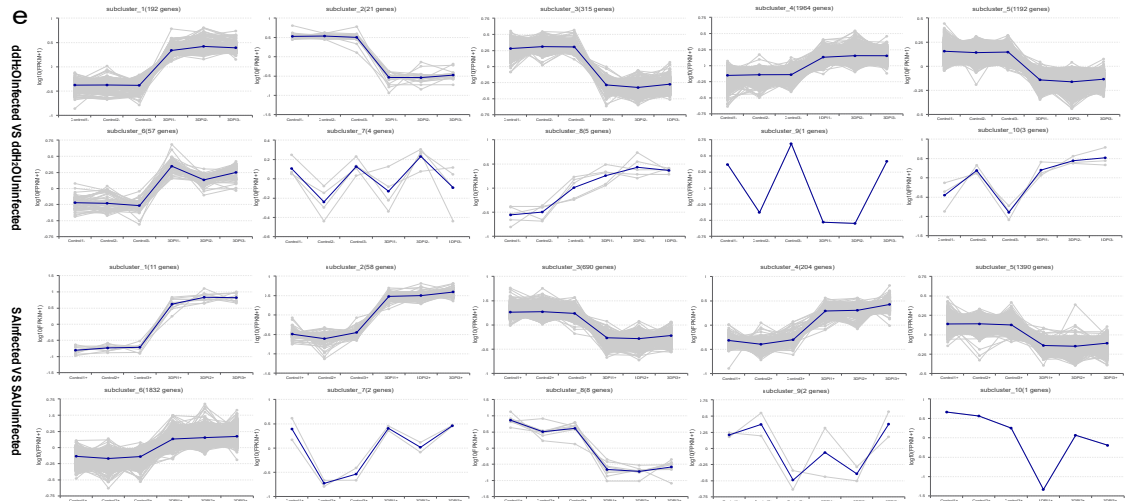

Supplement: Supplementary file 3 — Additional file 3 : Figure S3. DEGs in ddH2O-treated and SA-treated leaves. (a) The number of DEGs up- or downregulated at 3 dpi in both groups. (b) Heatmap generated from the DEGs from RNA-Seq analysis comparing the fold changes in gene expression during the infection stage. Heatmap colours represent gene expression fold-change levels based on the provided colour key scale; red = upregulated expression, blue = downregulated expression, and white = no change in expression. (c) Venn diagrams of the number of DEGs between ddH2O-treated and SA-treated groups. (d) Venn diagram showing a cross-comparison of the up- and downregulated genes from both groups. (e) Cluster analysis of DEGs in the ddH2O-treated and SA-treated groups based on the BIRCH method. [file 12870_2020_2353_MOESM3_ESM.pdf]

a

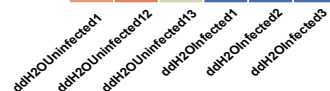

## b

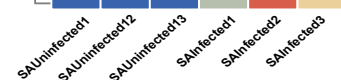

Supplement: Supplementary file 5 — Additional file 5 : Figure S5. Hierarchical clustering heatmap for DEGs in the ddH2OInfected/ddH2OUninfected and SAInfected/SAUninfected. (a) Cluster analysis of DEGs involved in plant hormone signalling between the ddH2OInfected/ddH2OUnifected; (b) cluster analysis of DEGs involved in plant hormone signalling between the SAInfected/SAUninfected. Differences are highlighted in blue (downregulation) and red (upregulation). [file 12870_2020_2353_MOESM5_ESM.pdf]

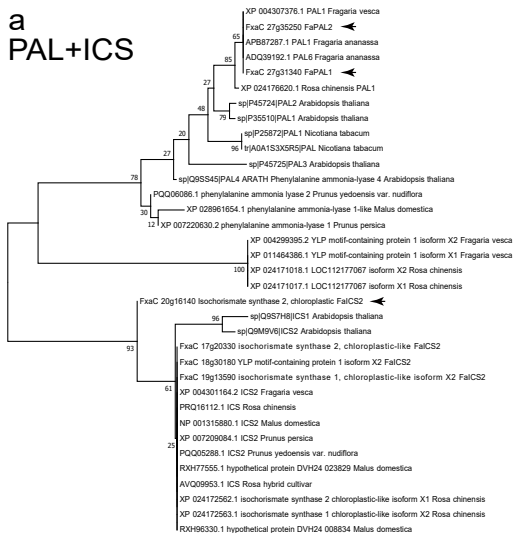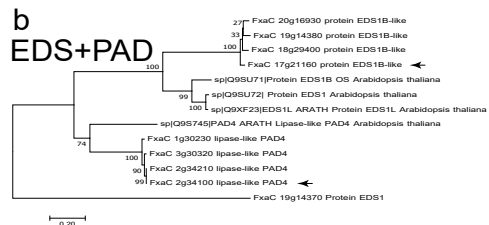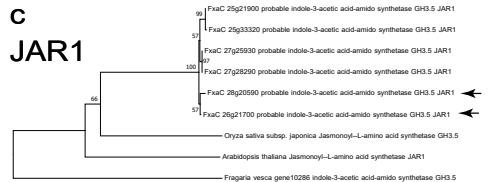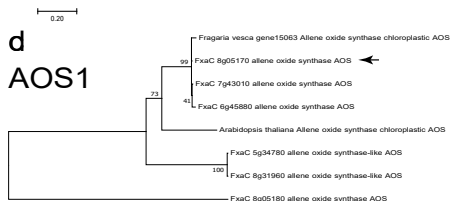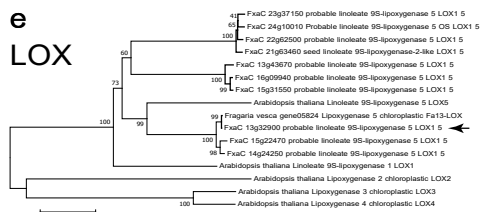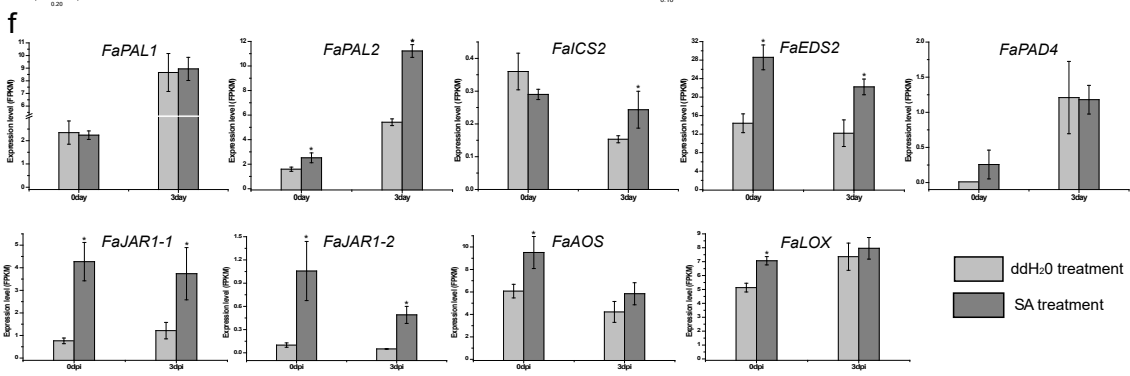

Supplement: Supplementary file 6 — Additional file 6 : Figure S6. Identification of putative genes involved in SA biosynthesis and JA biosynthesis in strawberry leaves infected by P. aphanis. The phylogenetic relationships existing between the strawberry PAL plus ICS (a), EDS1 plus PAD4 (b), JAR1 (c), AOS1 (d), and LOX (e) proteins used in this study (black arrow) with their closest homologues. Numbers at branches indicate posterior probabilities and bootstrap percentages based on 1000 replicates. (f) Changes in relative expression of FaPAL, FaICS2, FaEDS1, FaPAD4, FaJAR, FaAOS and FaLOX genes at 0 dpi and 3 dpi in both groups. The values were normalized against controls and correspond to the mean of three biological replicates ± S.E. Asterisks indicate significant differences (* p < 0.05). PAL, phenylalanine ammonia-lyase; ICS, isochorismate Synthase; EDS2, enhanced disease susceptibility2; PAD4, phytoalexin deficient 4; JAR1, JA-amino acid synthetase1; AOS, allene oxide synthase; LOX, lipoxygenase. [file 12870_2020_2353_MOESM6_ESM.pdf]
