# Supplementary material for: Salicylic acid-primed defence response in octoploid strawberry ‘Benihoppe’ leaves induces resistance against Podosphaera aphanis through enhanced accumulation of proanthocyanidins and upregulation of pathogenesis-related genes
Source: BMC Plant Biol. 2020 Apr 8;20:149. doi: 10.1186/s12870-020-02353-z (PMC7140339; doi:10.1186/s12870-020-02353-z)
Supplement: Supplementary file 4 — Additional file 4 : Figure S4. (a) Enriched Gene Ontology (GO) biological processes in ddH2OInfected/ddH2OUninfected. (b) Enriched Gene Ontology (GO) biological processes in SAInfected/SAUninfected. The y-axis represents enriched GO processes (false discovery rate < 0.05). The x-axis indicates the total number of genes annotated to each GO process. Red and blue sections represent downregulated and upregulated genes, respectively. (c) KEGG enrichment pathways (Top 20) for ddH2OInfected vs ddH2OUninfected. (d) KEGG enrichment pathways (Top 20) for SAInfected vs SAUninfected. The rich factor indicates the degree of enrichment. The colour and size of the dots indicate the range of the q-value and gene number, respectively. [file 12870_2020_2353_MOESM4_ESM.pdf]

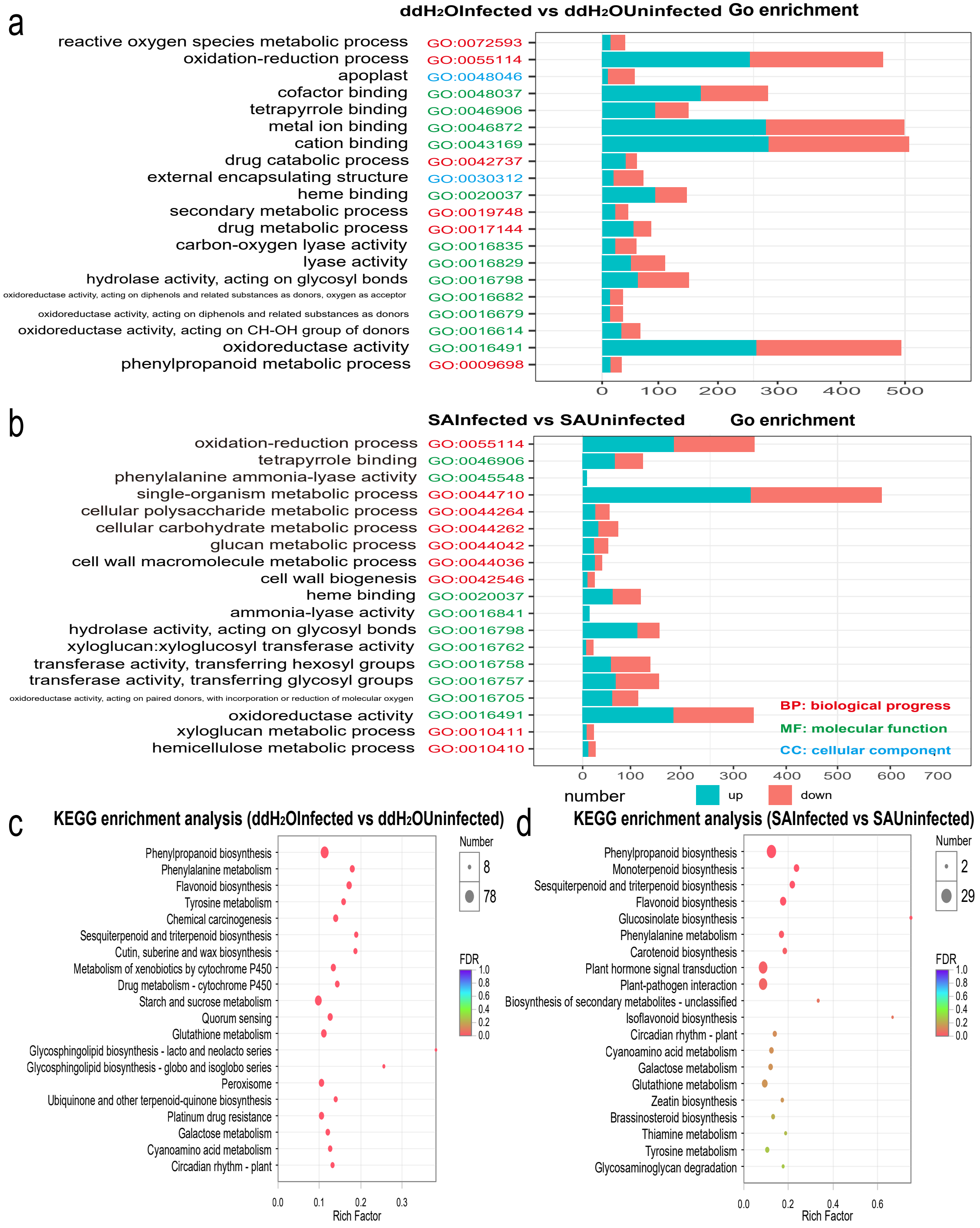
a
ddH2OInfected vs ddH2OUninfected Go enrichment
reactive oxygen species metabolic process GO:0072593
oxidation-reduction process GO:0055114
apoplast GO:0048046
cofactor binding GO:0048037
tetrapyrrole binding GO:0046906
metal ion binding GO:0046872
cation binding GO:0043169
drug catabolic process GO:0042737
external encapsulating structure GO:0030312
heme binding GO:0020037
secondary metabolic process GO:0019748
drug metabolic process GO:0017144
carbon-oxygen lyase activity GO:0016835
lyase activity GO:0016829
hydrolase activity, acting on glycosyl bonds GO:0016798
oxidoreductase activity, acting on diphenols and related substances as donors, oxygen as acceptor GO:0016682
oxidoreductase activity, acting on diphenols and related substances as donors GO:0016679
oxidoreductase activity, acting on CH-OH group of donors GO:0016614
oxidoreductase activity GO:0016491
phenylpropanoid metabolic process GO:0009698
0 100 200 300 400 500
b
SAInfected vs SAUninfected Go enrichment
oxidation-reduction process GO:0055114
tetrapyrrole binding GO:0046906
phenylalanine ammonia-lyase activity GO:0045548
single-organism metabolic process GO:0044710
cellular polysaccharide metabolic process GO:0044264
cellular carbohydrate metabolic process GO:0044262
glucan metabolic process GO:0044042
cell wall macromolecule metabolic process GO:0044036
cell wall biogenesis GO:0042546
heme binding GO:0020037
ammonia-lyase activity GO:0016841
hydrolase activity, acting on glycosyl bonds GO:0016798
xyloglucan:xyloglucosyl transferase activity GO:0016762
transferase activity, transferring hexosyl groups GO:0016758
transferase activity, transferring glycosyl groups GO:0016757
oxidoreductase activity, acting on paired donors, with incorporation or reduction of molecular oxygen GO:0016705
oxidoreductase activity GO:0016491
xyloglucan metabolic process GO:0010411
hemicellulose metabolic process GO:0010410
BP: biological progress
MF: molecular function
CC: cellular component
0 100 200 300 400 500 600 700
number
up
down
c
KEGG enrichment analysis (ddH2OInfected vs ddH2OUninfected)
Phenylpropanoid biosynthesis
Phenylalanine metabolism
Flavonoid biosynthesis
Tyrosine metabolism
Chemical carcinogenesis
Sesquiterpenoid and triterpenoid biosynthesis
Cutin, suberine and wax biosynthesis
Metabolism of xenobiotics by cytochrome P450
Drug metabolism - cytochrome P450
Starch and sucrose metabolism
Quorum sensing
Glutathione metabolism
Glycosphingolipid biosynthesis - lacto and neolacto series
Glycosphingolipid biosynthesis - globo and isoglobo series
Peroxisome
Ubiquinone and other terpenoid-quinone biosynthesis
Platinum drug resistance
Galactose metabolism
Cyanoamino acid metabolism
Circadian rhythm - plant
0.0 0.1 0.2 0.3
Rich Factor
Number
8
78
FDR
1.0
0.8
0.6
0.4
0.2
0.0
d
KEGG enrichment analysis (SAInfected vs SAUninfected)
Phenylpropanoid biosynthesis
Monoterpenoid biosynthesis
Sesquiterpenoid and triterpenoid biosynthesis
Flavonoid biosynthesis
Glucosinolate biosynthesis
Phenylalanine metabolism
Carotenoid biosynthesis
Plant hormone signal transduction
Plant-pathogen interaction
Biosynthesis of secondary metabolites - unclassified
Isoflavonoid biosynthesis
Circadian rhythm - plant
Cyanoamino acid metabolism
Galactose metabolism
Glutathione metabolism
Zeatin biosynthesis
Brassinosteroid biosynthesis
Thiamine metabolism
Tyrosine metabolism
Glycosaminoglycan degradation
0.0 0.2 0.4 0.6
Rich Factor
Number
2
29
FDR
1.0
0.8
0.6
0.4
0.2
0.0
